# Applying a Global Sensitivity Analysis Workflow to Improve the Computational Efficiencies in Physiologically-Based Pharmacokinetic Modeling

**DOI:** 10.3389/fphar.2018.00588

**Published:** 2018-06-08

**Authors:** Nan-Hung Hsieh, Brad Reisfeld, Frederic Y. Bois, Weihsueh A. Chiu

**Affiliations:** ^1^Department of Veterinary Integrative Biosciences, College of Veterinary Medicine and Biomedical Sciences, Texas A&M University, College Station, TX, United States; ^2^Chemical and Biological Engineering and School of Biomedical Engineering, Colorado State University, Fort Collins, CO, United States; ^3^INERIS, DRC/VIVA/METO, Verneuil en Halatte, France

**Keywords:** global sensitivity analysis, physiologically-based pharmacokinetic model, Bayesian, computational efficiency, parameter fixing

## Abstract

Traditionally, the solution to reduce parameter dimensionality in a physiologically-based pharmacokinetic (PBPK) model is through expert judgment. However, this approach may lead to bias in parameter estimates and model predictions if important parameters are fixed at uncertain or inappropriate values. The purpose of this study was to explore the application of global sensitivity analysis (GSA) to ascertain which parameters in the PBPK model are non-influential, and therefore can be assigned fixed values in Bayesian parameter estimation with minimal bias. We compared the elementary effect-based Morris method and three variance-based Sobol indices in their ability to distinguish “influential” parameters to be estimated and “non-influential” parameters to be fixed. We illustrated this approach using a published human PBPK model for acetaminophen (APAP) and its two primary metabolites APAP-glucuronide and APAP-sulfate. We first applied GSA to the original published model, comparing Bayesian model calibration results using all the 21 *originally* calibrated model parameters (OMP, determined by “expert judgment”-based approach) vs. the subset of *original influential* parameters (OIP, determined by GSA from the OMP). We then applied GSA to all the PBPK parameters, including those fixed in the published model, comparing the model calibration results using this *full set* of 58 model parameters (FMP) vs. the *full set influential* parameters (FIP, determined by GSA from FMP). We also examined the impact of different cut-off points to distinguish the influential and non-influential parameters. We found that Sobol indices calculated by eFAST provided the best combination of reliability (consistency with other variance-based methods) and efficiency (lowest computational cost to achieve convergence) in identifying influential parameters. We identified several originally calibrated parameters that were not influential, and could be fixed to improve computational efficiency without discernable changes in prediction accuracy or precision. We further found six previously fixed parameters that were actually influential to the model predictions. Adding these additional influential parameters improved the model performance beyond that of the original publication while maintaining similar computational efficiency. We conclude that GSA provides an objective, transparent, and reproducible approach to improve the performance and computational efficiency of PBPK models.

## Introduction

Physiologically-based pharmacokinetic (PBPK) modeling plays a critical role in the fields of predictive toxicology and pharmacology (Reisfeld and Mayeno, 2012; Chen et al., 2015). By including the physiological structures of organisms and the physiochemical properties of chemicals, PBPK models provide a quantitative description of the pharmacokinetic processes such as absorption, distribution, metabolism, and excretion, and can be used to investigate mechanistic processes, evaluate hypotheses, and guide experiment design. They can also help reduce animal testing through their ability to simulate and predict bio-distribution of target tissue dose of the parent chemicals and metabolites.

In toxicology and pharmacology, toxicokinetic or PBPK models are essential tools to evaluate the variability in chemical or drug concentrations among individuals in the target population (Jones and Rowland-Yeo, 2013; Ring et al., 2017). These models are particularly useful in helping to design safer treatments and/or assessing risks for sensitive populations, either for pharmaceuticals or environmental contaminants (Liao et al., 2010). PBPK models usually have dozens of parameters. This complexity poses a challenge in parameter estimation (Slob et al., 1997; Yates, 2006; Garcia et al., 2015; Wendling et al., 2015) and precludes the application of standard frequentist algorithms used for traditional PK analyses. Moreover, unlike traditional compartmental models, PBPK model parameters are not uniquely identifiable based on PK data alone (Yates, 2006). The usual solution to calibrate a PBPK model is to fix the “felt to be known” model parameters and optimize only a small subset of parameters (Peters, 2008; Lyons et al., 2013). However, this “expert judgment”-based approach lacks statistical rigor.

Bayesian statistics provides a rational way to address the identifiability issue in PBPK models through the use of informative prior knowledge in parameter distribution (Gelman et al., 1996, 2013; Chiu et al., 2014). Prior knowledge can be updated with informative data through a likelihood function, resulting in a so-called *posterior distribution* reflecting an updated estimate of each parameter. For population PBPK models, the use of a hierarchical Bayesian approach allows PBPK model parameters to be estimated both at the individual and the overall population level. In general, Markov chain Monte Carlo (MCMC) is used to estimate these posterior distributions. However, the determination of the posterior distribution for all parameters in complex dynamic models is very challenging owing to the model nonlinearity and interactions among multiple parameters. In PBPK models, nonlinear biological processes, such as enzyme saturation and interactions between physiological parameters, increase the computational difficulty in the Bayesian calibration process. Non-identifiable parameters can further increase the computational burden of Bayesian analysis, and therefore reduce the computational efficiency. Additionally, currently implemented Bayesian numerical algorithms for PBPK modeling often have challenges in convergence with acceptable computational times due to the need to solve systems of coupled ordinary differential equations (Wendling et al., 2015).

Sensitivity analysis (SA) provides a means to evaluate the influence of model parameters and identify non-influential parameters that can be fixed with minimal effect on model output (Ratto et al., 2007; Zhang et al., 2015). Sensitivity analyses can generally be grouped into two categories: local and global (Pianosi et al., 2016). Typically, local SA uses the one-at-a-time sampling method to examine the uncertainty of parameter impacts on model output. This straightforward method has high efficiency in computational analysis, but neglects the interactions and simultaneous variations in various input parameters and may give the misleading results. Unlike local SA, global SA (GSA) calculates the contribution from the variety of all model parameters, including both single parameter effects and multiple parameter interactions. This approach has been widely applied to biological models (McNally et al., 2011; Boas et al., 2015; Loizou et al., 2015; Lumen et al., 2015). For example, McNally et al. (2011) provided a GSA workflow for PBPK models that begins with preliminary screening from elementary effect (EE)-based Morris method to eliminate (i.e., fix at nominal values) the parameters with negligible impact on the model output, and then used variance-based GSA to determine the influential parameters in the PBPK model. GSA thus provided an algorithm to simplify the computational approach while maintaining accuracy and precision, thereby improving computational efficiency.

Our study hypothesis is that *GSA can provide a systematic method to ascertain which PBPK model parameters have negligible influence on model outputs and can be fixed to improve computational speed in Bayesian parameter estimation with minimal bias*. Although GSA offers many advantages compared to local SA, only a few applications in PBPK modeling have been published. For instance, a previous study for a PBPK model of m-Xylene demonstrated that parameters identified by GSA as having little influence had similar posterior distributions to those when all parameters were calibrated using the Bayesian approach (McNally et al., 2012). Here, we extend this approach in a new case study using a more complex model: a PBPK model for acetaminophen (APAP) and its conjugated metabolites. We used this case study to answer four key questions: (1) What is the relative computational efficiency/rate of convergence of various GSA algorithms? (2) Do different algorithms give consistent results as to direct and indirect parameter sensitivities? (3) Can we identify non-influential parameters that can be fixed in a Bayesian PBPK model calibration while achieving similar degrees of accuracy and precision? (4) Does fixing parameters using “expert judgment” lead to unintentional imprecision or bias?

We examined questions (1) and (2) by applying four different GSA algorithms to the PBPK model. For question (3), we compared the results of MCMC simulations of the PBPK model with and without fixing non-influential parameters. We applied each of these analyses to the PBPK model using (a) the original set of 21 model parameters (OMP), calibrated in the previously published model, which also included 37 parameters fixed by expert judgment, resulting in the influential subset of these original parameters (OIP, determined by the GSA approach); and (b) the full set of 58 model parameters (FMP) including those previously fixed, resulting in the influential subset of these parameters (FIP, determined by the GSA approach). Thus, question (4) was examined by comparing the results obtained from OMP, OIP, FMP, and FIP.

## Materials and methods

### APAP-PBPK model, parameters, and data

Our analysis made use of our previously developed PBPK model that describes the ADME of APAP and its conjugated metabolites, APAP-glucuronide (APAP-G) and APAP-sulfate (APAP-S) in humans (Zurlinden and Reisfeld, 2016, 2017). All model details such as structure, parameters, and state variables are detailed in this publication, but in brief, the model equations were constructed based on chemical mass balances, assuming blood-flow limited transport and human physiological properties. Model compartments included fat, muscle, liver, gastrointestinal (GI), and kidney, with remaining tissues lumped into either rapidly- and slowly-perfused compartments. Standard Michaelis-Menten saturation kinetics were used to quantify the process of APAP metabolism for APAP-G and APAP-S in the liver, and were parameterized by the Michaelis constant Km and maximum metabolism rate Vmax. The original PBPK model was able to predict the distribution of these chemicals in target tissues adequately. Distributions for parameter priors were derived from literature values and were assumed to be uniform or truncated normal distributions under the log-transformed scale (Price et al., 2003; Chiu et al., 2009; Zurlinden and Reisfeld, 2016). These parameter probability distributions are summarized in Table 1. Parameters that did not have measured values were assumed to follow a biologically-plausible distribution for sensitivity analysis and model calibration. We further assumed that the parameters in the PBPK model were independently distributed. Therefore, all parameters could be sampled independently, which is required for the GSA algorithms we applied (see Li et al., 2010 for a method that can incorporate correlated inputs). This assumption is likely to not be strictly true, for instance with physiological parameters, but recent work by Ring et al. (2017) using correlated physiological parameters suggests that such correlations have minimal impact on PBPK model predictions.

**Table 1:** Description of prior distributions for original and additional parameters^a^.

**Parameter**	**Description**	**Unit**	**Distribution**	**Mean or min**	**sdlog (z-score truncation) or max**
**ORIGINAL PARAMETERS**					
Tg	Gatric emptying time constant	*h*	LN	0.23	0.5 (±3)
Tp	GI perfusion time constant	*h*	LN	0.033	0.5 (±3)
CYP_Km	Cytochrome P450 metabolism, Km	μM	LN	130	1 (±1)
CYP_VmaxC	Cytochrome P450 metabolism, VMax	μmole/h·BW^0.75^	U	0.14	2900
SULT_Km_apap	Sulfation pathway acetaminophen, Km	μM	LN	300	1 (±1)
SULT_Ki	Sulfation pathway substrate inhibition, Ki	μM	LN	526	0.5 (±2)
SULT_Km_paps	Sulfation pathway PAPS Km	–	LN	0.5	0.5 (±2)
SULT_VmaxC	Sulfation pathway acetaminophen, Vmax	μmole/h·BW^0.75^	U	1	3.26e6
UGT_Km	Glucronidation pathway acetaminophen, Km	μM	LN	6.0e3	1 (±1)
UGT_Ki	Glucronidation pathway substrate inhibition, Ki	μM	LN	5.8e4	0.5 (±2)
UGT_Km_GA	Glucronidation pathway GA Km	–	LN	0.5	0.5 (±2)
UGT_VmaxC	Glucronidation pathway acetaminophen, Vmax	μmole/h·BW^0.75^	U	1	3.26e6
Km_AG	APAP-G hepatic transporter Km	μM	LN	1.99e4	0.3 (±3)
Vmax_AG	APAP-G hepatic transporter Vmax	μmole/h	U	1.09e3	3.26e6
Km_AS	APAP-S hepatic transporter Km	μM	LN	2.99e4	0.22 (±3)
Vmax_AS	APAP-S hepatic transporter Vmax	μmole/h	U	1.09e3	3.26e6
kGA_syn	UDPGA synthesis	1/h	U	1	4.43e5
kPAPS_syn	PAPS synthesis	1/h	U	1	4.43e5
CLC_APAP	APAP clearance	L/h·BW^0.75^	U	2.48e-3	2.718
CLC_AG	APAP-G clearance	L/h·BW^0.75^	U	2.48e-3	2.718
CLC_AS	APAP-S clearance	L/h·BW^0.75^	U	2.48e-3	2.718
**ADDITIONAL PARAMETERS**					
QCC	Cardiac output	L/h·BW^0.75^	LN	16.2	0.2 (±4)^b^
VFC	Fraction volume of fat	–	LN	0.214	0.45 (±2)^b^
VKC	Fraction volume of kidney	–	LN	0.0044	0.17 (±2)^b^
VGC	Fraction volume of gut	–	LN	0.0144	0.08 (±2)^b^
VLC	Fraction volume of liver	–	LN	0.0257	0.23 (±2)^b^
VMC	Fraction volume of muscle	–	LN	0.4	0.34 (±2)^c^
VBLAC	Fraction volume of arterial blood	–	LN	0.0243	0.12 (±2)^b^
VBLVC	Fraction volume of venous blood	–	LN	0.0557	0.12 (±2)^b^
VSC	Fraction volume of slowly perfused tissue	–	LN	0.185	0.34 (±2)^b^
QFC	Fractional blood flow of fat	–	LN	0.052	0.46 (±2)^b^
QKC	Fractional blood flow of kidney	–	LN	0.175	0.18 (±2)^b^
QGC	Fractional blood flow of gut	–	LN	0.181	0.45 (±2)^b^
QLBC	Fractional blood flow of hepatic artery	–	LN	0.046	0.12 (±2)^b^
QMC	Fractional blood flow of muscle	–	LN	0.191	0.32 (±2)^c^
QSC	Fractional blood flow of fat	–	LN	0.14	0.35 (±2)^b^
BP_APAP	Blood and plasma ratio	–	LN	0.9	0.4 (±3)^b^
PF_APAP	APAP partition coefficient of fat	–	LN	0.447	
PG_APAP	APAP partition coefficient of gut	–	LN	0.907	
PK_APAP	APAP partition coefficient of kidney	–	LN	0.711	
PL_APAP	APAP partition coefficient of liver	–	LN	0.687	
PM_APAP	APAP partition coefficient of muscle	–	LN	0.687	
PR_APAP	APAP partition coefficient of rapidly perfused tissues	–	LN	0.676	
PS_APAP	APAP partition coefficient of slowly perfused tissues	–	LN	0.606	
PF_AS	APAP-S partition coefficient of fat	–	LN	0.088	
PG_AS	APAP-S partition coefficient of gut	–	LN	0.297	
PK_AS	APAP-S partition coefficient of kidney	–	LN	0.261	
PL_AS	APAP-S partition coefficient of liver	–	LN	0.203	
PM_AS	APAP-S partition coefficient of muscle	–	LN	0.199	
PR_AS	APAP-S partition coefficient of rapidly perfused tissues	–	LN	0.207	
PS_AS	APAP-S partition coefficient of slowly perfused tissues	–	LN	0.254	
PF_AG	APAP-S partition coefficient of fat	–	LN	0.128	
PG_AG	APAP-G partition coefficient of gut	–	LN	0.436	
PK_AG	APAP-G partition coefficient of kidney	–	LN	0.392	
PL_AG	APAP-G partition coefficient of liver	–	LN	0.321	
PM_AG	APAP-G partition coefficient of muscle	–	LN	0.336	
PR_AG	APAP-G partition coefficient of rapidly perfused tissues	–	LN	0.364	
PS_AG	APAP-G partition coefficient of slowly perfused tissues	–	LN	0.351	

a Original parameters and nominal value of all additional parameters were adapted from Zurlinden and Reisfeld (2016).

b Parameter uncertainty adapted from Chiu et al. (2009) with truncation at z-scores of ±z (log-transformed mean ± z × log-transformed SD).

c *Parameter uncertainty adapted from Price et al. (2003) with truncation at z-scores of ±z (log-transformed mean ± z × log-transformed SD)*.

Available pharmacokinetic data from published clinical studies of APAP and its metabolites, also previously reported, were used for model calibration. Studies involved a single oral dose ranging from 325 mg to about 1,400 mg (20 mg/kg). The sources of the human experimental data and their corresponding dose levels are in Table S1. Prior PBPK model predictions were checked as to their coverage of the calibration data (Figure S1). Specifically, the prior distributions for the model parameters were sampled by Monte Carlo, and the simulations run for each random parameter set.

### GSA algorithms and approach

#### Morris screening

The Morris method (Morris, 1991) is an extension of the traditional one-at-a-time (OAT) approach, but over multiple points in the multi-dimensional parameter spaces to provide global OAT analysis through a number of sampling trajectories. Beginning at a randomly sampled point, each PBPK parameter was sampled along a grid to create a trajectory through the parameter space. According to the standard practice for this method, all parameters were re-scaled with the minimum scaled to zero, the maximum scaled at 1, and the re-scaled parameter assumed to follow the uniform distribution in [0, 1]. For the parameters with lognormal distributions, the rescaling was performed on the natural log-transformed parameters. These parameters were truncated at z-scores of ±2- to 4-fold standard deviations (shown in parentheses in Table 1), and those limits used as the minimum and maximum. Outputs from the Morris method include the mean OAT sensitivities μ and its standard deviation σ of the distribution, which represents the overall influence and the interactions/non-linear effects on the PBPK model output, respectively. The Morris σ also represent the variability throughout the parameter space. Our study adopted the more advanced EE-based Morris method that was developed by Campolongo et al. (2007), which improved both the effectiveness and reliability of the sensitivity measures (adding the mean absolute value of distribution of the OAT sensitivities, denoted μ^*^) as well as the efficiency of the sampling strategy (generating a large number of proposed sampling trajectories with Latin Hypercube sampling, and selecting a subset with the highest diversity across parameter space). The design generated random starting point for each parameter in the specific range and then moved one at a time in a random order. The minimum and maximum value for each parameter from the prior distribution in Table 1 were used to define the sampling range. To compare the result of the Morris screening with the Sobol index, we normalized the Morris index for each independent output, so the maximum index for each of the three different compounds at a specific given time *t* was 1.

#### Variance-based sensitivity analysis

More sophisticated GSA methods include so-called “variance-based” methods that aim to partition variance in the model output among both individual parameter influences and parameter interactions. All the variance-based GSA methods we used are based on calculating so-called *Sobol sensitivity indices*, specifically “Main” (first-order), “Total” (total-order) effect, and “Interaction” (their difference). The *main effect* is used to measure the contribution proportion of a specific parameter to the output variation and reflect the expected reduction in the output variance if the parameter were known precisely. Therefore, the magnitude of the main-effect can be used to help determine if the specific parameter can be fixed. The *interaction effect* is used to measure the contribution proportion of parameter interactions to the output variation, which quantifies the effect of interactions of two or more parameters on the output. The *total effect* characterizes the effect of the specific parameter and the results of its interaction with all other parameters on the variation of the model output. It represents the expected amount of output variance that would remain unexplained (residual variance) if only that variable were left free to vary over its range. Previous studies have used the extended Fourier amplitude sensitivity test (eFAST) (Saltelli et al., 1999) method to calculate the Sobol sensitivity indices, but alternative algorithms using different sampling schemes are also available (Saltelli et al., 2010). Variance-based methods are more computationally intensive than the Morris method, so in addition to using eFAST, this study investigated two other variance-based methods to assess computational efficiency. Both these additional methods are Monte Carlo-based (Jansen, 1999; Owen, 2013), making use of two or three independent samples to estimate the Sobol indices.

#### Distinguishing influential and non-influential parameters

Separate sensitivity indices can be produced for each output measurement, so for pharmacokinetic data, there are indices not only for different measurements (plasma concentrations of APAP, APAP-S, and APAP-G), but also as a function of time. All model outputs were transformed to logarithmic scale to be consistent with the lognormal likelihood function used for Bayesian model calibration (see section MCMC Simulations, below). This also avoids excessive scale-related effects, such as due to data being in different units. The logarithmic transformation may not be appropriate for measurements near the limit of detection or the numerical error tolerance of the prediction simulations, neither of which was an issue in this case. Because data may have been taken at different sampling time-points across a group of subjects, it is often difficult to summarize the overall impact of a parameter on the model calibration. Therefore, we selected representative time points of 0.5-, 1-, 1.5-, 2-, 4-, 6-, 8-, and 12-h post intake as the SA time points, which included the entire time-period of detectable concentrations in plasma in the clinical data. For each parameter, we then used the maximum sensitivity index across all data points (APAP, APAP-S, APAP-G, each at time points 0.5–12 h) as the indicator of sensitivity. After that, we set a cut-off point as a benchmark to distinguish the influential and non-influential parameters (see section Selection of influential/non-influential parameters, below). This choice was made because the only way a parameter can be labeled “non-influential,” and thus a candidate for fixing, is if all of the sensitivity indices for that parameters are “small.” *Thus, the separation between “influential” and “non-influential” was driven by the most sensitive output for each parameter*.

#### Convergence of sensitivity index

All GSA results required the model to be evaluated in a finite sample of the overall parameter space, so convergence was an important metric to monitor. By convergence, we mean that the result of sensitivity index is similar across replications under the same sample size by using a bootstrap approach (for Jansen and Owen methods) or a random phase-shift (for eFAST). Both methods can generate confidence interval for the sensitivity indices. The number of model evaluations is based on a sample size *n* and number of input factors *p*, and it is notable that the total number of model evaluations is different between Morris (*n* × (*p*+1)), eFAST (*n* × *p*), Jansen (*n* × (*p*+2)), and Owen (*n* × (3*p*+2)). We estimated the convergence value of sensitivity indices for each parameter using the approach proposed by Sarrazin et al. (2016). This method quantitatively assesses the convergence by computing the range of 95% confidence intervals (from bootstrap or random phase shift) of the total sensitivity indices for each parameter across all data points. For our analysis, the maximum estimated convergence values in all model parameters were used as this metric. A confidence interval close to zero represents the parameter approaching convergence. For Sobol indices, we considered the threshold value as 0.1 (95% confidence interval less than 0.1) for the main and total effect to determine an acceptable result based on the suggestion from a previous study (Herman et al., 2013). For Morris indices, we normalized the index by maximum estimation and set the threshold as 10% of maximum estimation across model parameters for the specific compound at time *t*. We then used the maximum convergence index across all datasets (each parameter for each output variable) as representative. Therefore, the convergence index less than 0.1 (or 10% of maximum) was used as a measure of acceptable convergence.

#### Comparison of GSA sensitivity indices

To investigate whether the different algorithms give consistent results as to direct and indirect parameter sensitivities, we used the Pearson and Spearman correlation coefficients to assess the consistency of the main effects (Morris μ^*^ and Sobol main effect) and interaction effects (σ, and Sobol interaction effect) among each tested parameter using its maximum sensitivity index.

#### Selection of influential/non-influential parameters

For the EE-based Morris method, a cut-off point was set at 0.1 for both the normalized μ^*^ and σ for each dataset. If either the normalized μ^*^ or the normalized σ is greater than 0.1, the parameter can be considered “influential”. For Sobol indices, we compared cut-off values of 0.01 and 0.05 for both the total and interaction effects. We defined the parameter as influential if the estimated main effect *or* interaction was greater than the cut-point criteria. The interpretation of these measures is that parameters with smaller indices contribute less than 1 or 5% of the variance in the output, and thus would be considered “non-influential” (Zhang et al., 2015).

### MCMC simulations

In our previous study (Zurlinden and Reisfeld, 2016), only 21 metabolism and elimination-related parameters were used to perform the statistical model calibration and validation with experiment data. The other 37 PBPK model parameters were fixed based on the “expert judgment” that the clearance-related parameters were the most influential. Here, we evaluated global parameter sensitivity both for the OMP alone, as well as the FMP. As a benchmark, the Bayesian model calibration for a PBPK model was initially performed for both the OMP and FMP, recording baseline values for computational time and model performance. After that, the dimensionality reductions were made based on the cutoffs described above; Bayesian calibration was then performed for the reduced “influential” parameter sets (OSF and FIP), and the results compared to those using the OMP and FMP. Four independent Markov chains were run for each Bayesian analysis. As in our previous similar Bayesian analyses, we used the Gelman-Rubin potential scale reduction factor to assess whether different independent Markov chains had converged to a consistent distribution (Gelman and Rubin, 1992). For converged simulations, we used the predicted (log10-transformed) residuals from the calibration result to compare the model performance. The log-likelihood function was used to assess the goodness-of-fit of the model to the data (Woodruff and Bois, 1993), defined as

$$LL=\sum_{i=1}^{N}-\frac{1}{2}\cdot \frac{{\left(y_{i}-{\hat{y}}_{i}\right)}^{2}}{S_{j\left[i\right]}^{2}}-\frac{1}{2}ln\left(\begin{array}{ll} 2\pi & s_{j\left[i\right]}^{2} \end{array}\right)$$

where *N* is the total number of the data points used in model calibration; *y*_*i*_ and ŷ_*i*_ are the experimental observed and model predicted value (log-transformed), respectively; $s_{j}^{2}$ is the variance for data type *j* (i.e., APAP and each metabolite have separate variances), and *j*[*i*] is the data type of data point *i*.

This function aggregates the likelihood of experimental data value and corresponding model-predicted value across all experimental data including different time points and output variables. All individual likelihood functions were lognormal, which was equivalent to transforming model predictions to a logarithmic scale, as was done for GSA.

### Software and computing platform

Statistical analysis and results visualization of this study were carried out in R v.3.4. GSA was performed with the R “sensitivity” package v.1.15 (Pujol et al., 2017). We checked the convergence and compared the test result of Morris and eFAST method by using the Python v2.7.6 software package “SALib” v.1.0.3 (Herman and Usher, 2017). The MCMC simulations and output predictions were conducted using GNU MCSim v.5.6 (Bois, 2009). Necessary model parameter distributions were available within GNU MCSim, and Metropolis within Gibbs sampling (one component at a time) was used during simulation process. Aside from estimated of parameter posteriors, output from GNU MCSim also included the diagnostic information, such as the log-likelihood (LnData) at each iteration. Parallelized computation of the MCMC was performed within the 64-bit CentOS Linux distribution on a high-performance computing cluster at Texas A&M University, comprising machines with Intel Xeon 2.5GHz E5-2670 v2 (Ivy Bridge-EP) 10-core processors and 64 GB of RAM each. The inspection and comparison of the convergence results from the MCMC analyses were carried out using the R “boa” package v.1.15 (Smith, 2016). The computation of GSA was conducted on a Dell Optiplex 7040 desktop computer with Intel(R) Core(TM) i7-6700 CPU 3.4 GHz 16GB RAM.

## Results

The results of our analyses are organized along the four key questions described in the introduction.

### What is the relative computational efficiency/rate of convergence of various GSA algorithms?

**Figure 1:**
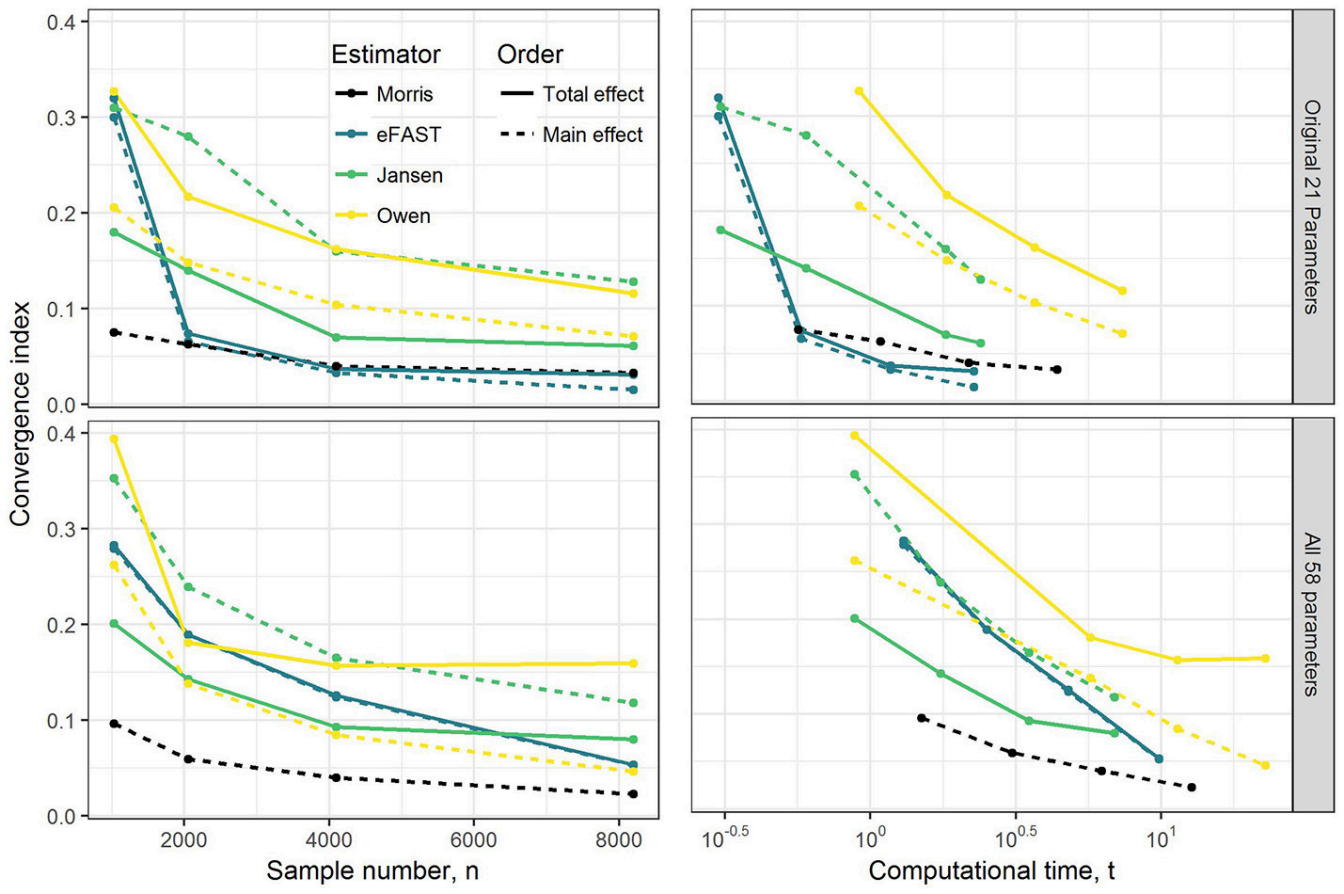
Illustration of the effect of GSA sampling number on convergence index and computational time (min). Note that to check convergence, the sample size has been increased up from 1,024 to 8,192 under OMP and FMP. Each estimated convergence index was based on the model evaluation that was generated from the sample number *n*.

Results of convergence analysis across all the GSA methods of Morris, eFAST, Jansen, and Owen are shown in Figure 1. In each case, the maximum index (i.e., combination of time-point, dataset, parameter, compound, and main vs. total effect that converges the slowest) is shown, along with the cost in terms of number of model evaluations and computational time. For the Morris screening method, the analysis with the small sample number of 1024 (resulting in 22,528 model evaluations) reached an acceptable converged result (convergence index < 0.1), which is not surprising, since Morris is an extension of a computationally-efficient local SA. Among the three variance-based Sobol indices, eFAST rapidly converged with a sample number of 2048 for the OMP and with 8192 (resulting in 475,136 model evaluations) for FMP. The alternative methods of Jansen and Owen did not converge, even up to a sample number of 8192. Overall, the Morris method provided the most efficient computational performance and convergence result, followed by eFAST.

### Do different algorithms give consistent results as to direct and indirect parameter sensitivities?

**Figure 2:**
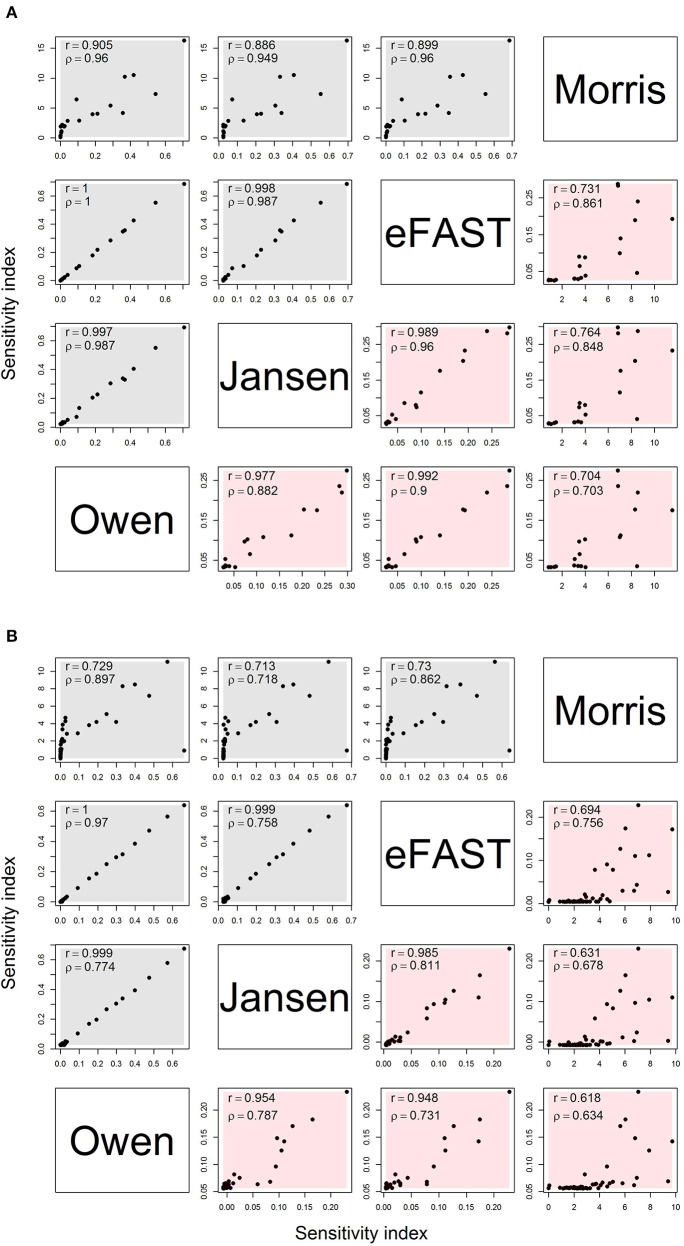
Correlation matrix for main (gray) and interaction (red) effects for the Morris, eFAST, Jansen, and Owen estimates by using the maximum sensitivity index for each parameter under **(A)** the OMP and **(B)** FMP. Both Pearson's r and Spearman's ρ are shown.

Figure 2 shows the comparison of sensitivity indices between the Morris method (sensitivities μ^*^ and non-linear/interacts effects σ) and variance-based global method (main and interaction) with the sample size of 8,192. For the OMP (Figure 2A), the variance-based Sobol indices showed a high correlation (*r* > 0.9) with each other. The Morris indices had the relatively lower correlation with variance-based indices. In addition, the rank correlation results were higher between Morris with Sobol indices than the product moment correlation. Therefore, the Morris indices could be used to preliminarily distinguish the non-influential parameters that were located at the lower level of the correlation plot. We also found that the correlation of the interaction had a lower range (*r*: 0.704–0.992) than the main effects (*r*: 0.886–1.000). The correlation plot for the Morris- and variance-based indices shows a “hockey stick” shape, suggesting that there are different correlation properties between “influential” and “non-influential” parameters. The FMP shows similar correlation properties for the sensitivity indices (Figure 2B). Compared with the OMP, the correlations of the interaction effects in FMP for variance-based indices were lower overall (*r*: 0.618–0.985). In addition, the number of highly influential parameters (approximately 10) were similar across all analysis.

### Can we identify “non-influential” parameters that can be fixed in a bayesian PBPK model calibration while achieving similar degrees of accuracy and precision?

Table 2 summarizes the sensitivity of model parameter under the OMP and FMP settings, respectively. The details of the GSA results are shown in Supplementary Materials.

**Table 2:**
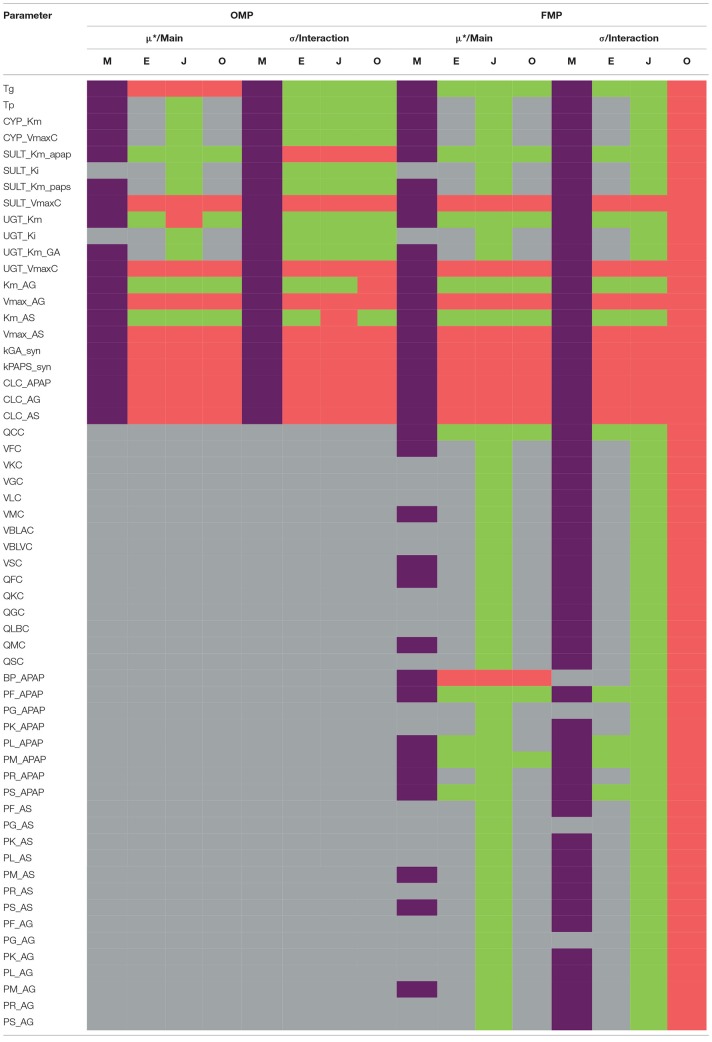
Parameter-specific sensitivity results for OMP and FMP using different GSA methods.

For the OMP, the Morris screening results indicated that only SULT_Ki and UGT_Ki should be grouped with the non-influential parameter, using the cut-off point of 0.1 for the main effect (no parameters could be deemed non-influential using the interaction; Figure S2). Here, all indices had been normalized with the same dataset, compound, and time point to make them more comparable. For the variance-based indices, for a cut-off point of 0.01, all of the OMP were classified as influential due to the estimated interaction effects being greater than the cut-off (Figure S3). Results generated from the Jansen method for the main effects were inconsistent with those for the eFAST and Owen methods, possibly due to a lack of convergence in the Jansen methods. Using a cut-off point of 0.05, between 11 and 14 of the OMP were considered influential, so the three methods gave more similar results (Figure S4). Because the eFAST approach had better convergence characteristics, we choose the 11 parameters (either main effect or interaction greater than 0.05) identified by this method as the original “influential” parameters (OIP) to use in the reduced-parameter MCMC analyses.

**Figure 3:**
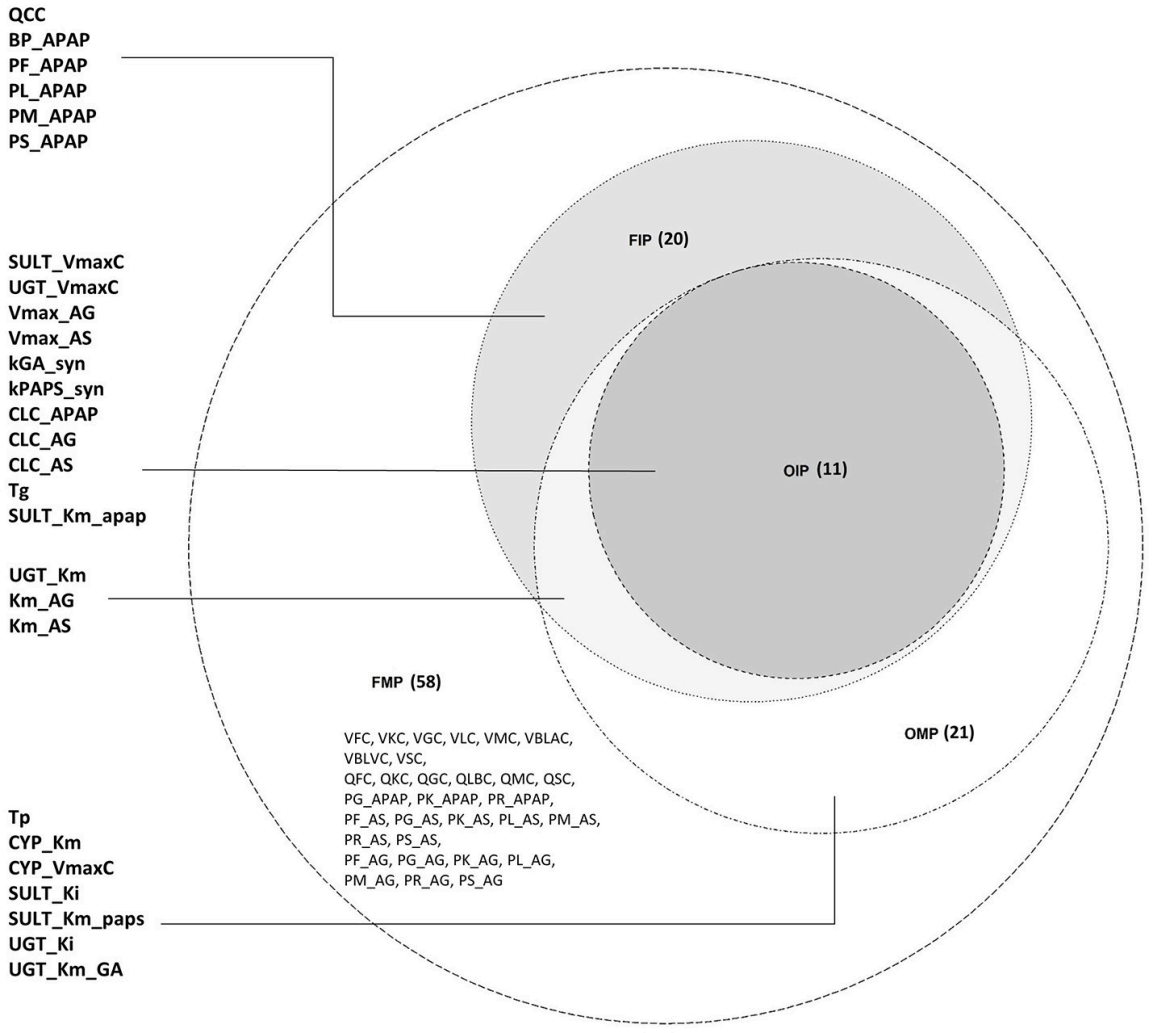
Venn diagram displaying the overlaps among the following four parameter sets: original model parameters, OMP; full set of model parameters, FMP; original influential parameters, using 0.05 as a cut-off point, OIP; full set of influential parameters, using 0.01 as a cut-off point, FIP.

For the FMP, the Morris screening result showed that only three additional parameters (PG_APAP, PG_AS, and PG_AG) that were fixed in the previous study were non-influential (Figure S5; Zurlinden and Reisfeld, 2016). In addition, for the variance-based methods, the Jansen and Owen methods showed all parameters to be influential, but these results had not fully converged. *This lack of convergence, along with the inconsistencies seen with the OMP, led us to focus on the eFAST method as representing the best balance among reliability, efficiency, and the ability to discriminate between influential and non-influential parameters*. For the cut-off point at 0.01, 20 parameters were identified as influential (FIP_01_). This included six parameters that were previously fixed in the previous study: QCC, BP_APAP, PF_APAP, PL_APAP, PM_APAP, and PS_APAP (Figure S6). Using a cut-off point of 0.05, 10 parameters were identified as “influential” (FIP_05_), among which one was previously considered to be a fixed parameter (BP_APAP) (Figure S7). In addition, BP_APAP had the main effects above the cut-off, and the interaction effects below. Figure 3 shows a Venn diagram that displays that the final determination of influential and non-influential parameter from eFAST in each dataset.

**Figure 4:**
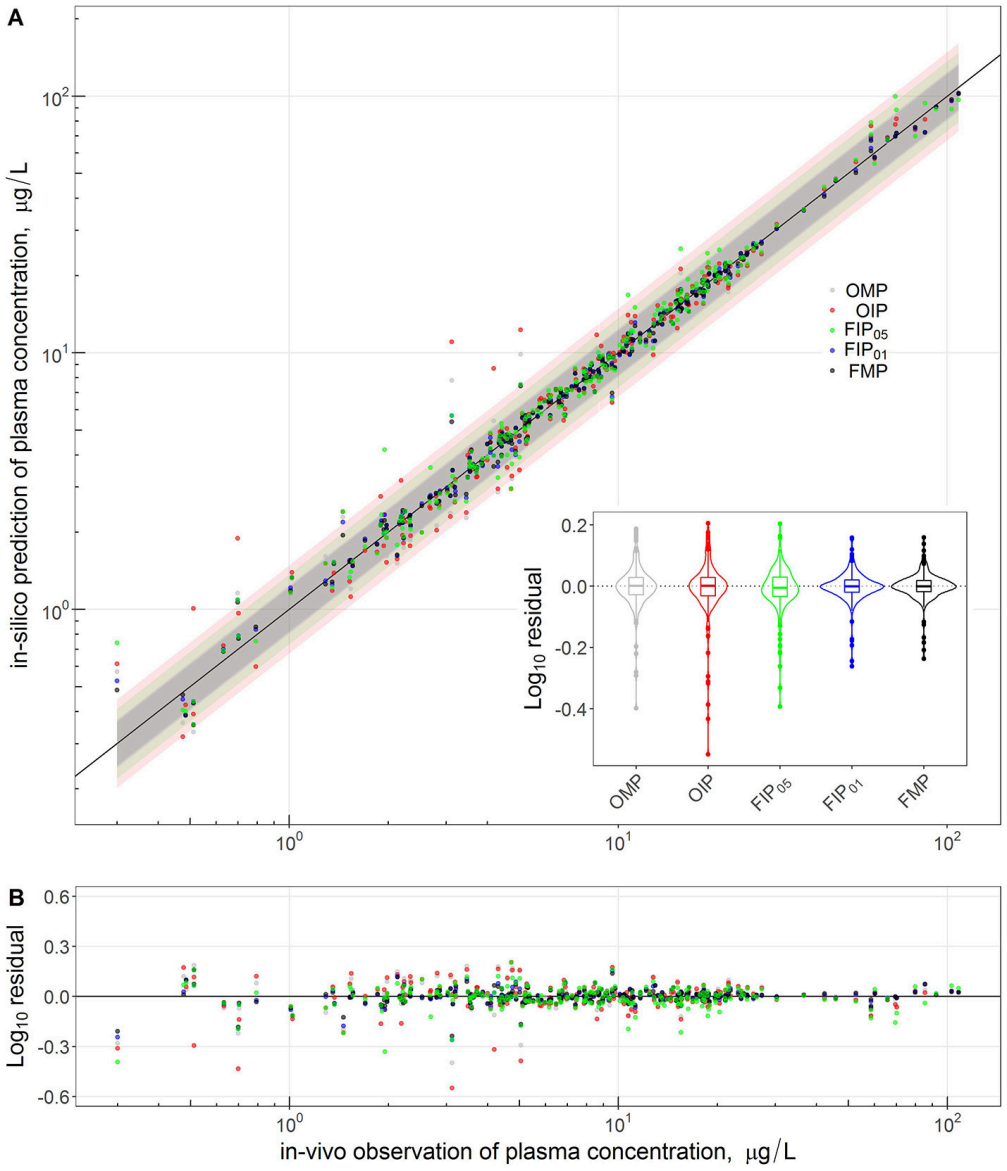
Comparison and global evaluation of the PBPK simulation results with residual properties estimation to determine the accuracy (residual median) and the precision (residual distribution) in model performance. The top **(A)** shows the relationships between experimental data (x-axis) and PBPK model predictions (y-axis) and 95% predicted interval for five model parameter settings. The bottom **(B)** shows the residuals from the predicted and experimental values to evaluate the accuracy and precision of model performance.

Figure 4 compares the overall global evaluation of model fits across all the alternative analyses: the OMP, FMP, OIP, FIP_01_, and FIP_05_. Figure 4A compares the observed data and model predictions, including the 95% prediction intervals (PIs). Figure 4B compares the residuals from the predicted result vs. experimental values to evaluate the accuracy and precision of model performance. The simulations had better predictions at higher concentrations (>10 μg/L). Based on estimated mean/median residuals and their distribution (the inset boxplot), we further found that the FIP_01_ simulation with 20 parameters had better accuracy and precision than the OMP simulations. Using the cut-off point at 0.05, the results using only “influential” parameters (FIP_05_) were similar to those using the OMP. Table 3 summaries the time-cost in GSA and MCMC analyses as the measurement of computational efficiency. *Overall, we found that restricting the MCMC simulations to the influential parameters can substantially reduce computational burden while showing little change in model performance*.

**Table 3:** Summary of parameter numbers and computational run times for GSA and MCMC.

		**OMP**	**FMP**
Number of parameters		21	58
MCMC run time (h)^a^		40.8 ± 0.18	104.6 ± 0.96
GSA-EE run time (h)^b^	Morris	0.009 ± 0.0002	0.04 ± 0.0004
GSA-Sobol run time (h)^c^	eFAST	0.07 ± 0.001	0.33 ± 0.001
	Jansen	0.13 ± 0.001	0.33 ± 0.004
	Owen	0.22 ± 0.001	0.99 ± 0.002
Sensitivity cut-off point > 0.05		OIP	FIP_05_
Number of influential parameters		11	10
MCMC run time (h)^a^		38.1 ± 0.07	24.8 ± 0.44
Sensitivity cut-off point > 0.01		(=OMP)	FIP_01_
Number of influential parameters		21	20
MCMC run time (h)^a^		40.8 ± 0.18	42.1 ± 0.29

a Number of iterations = 300,000.

b Number of samples = 1,024.

b *Number of samples = 8,192*.

**Figure 5:**
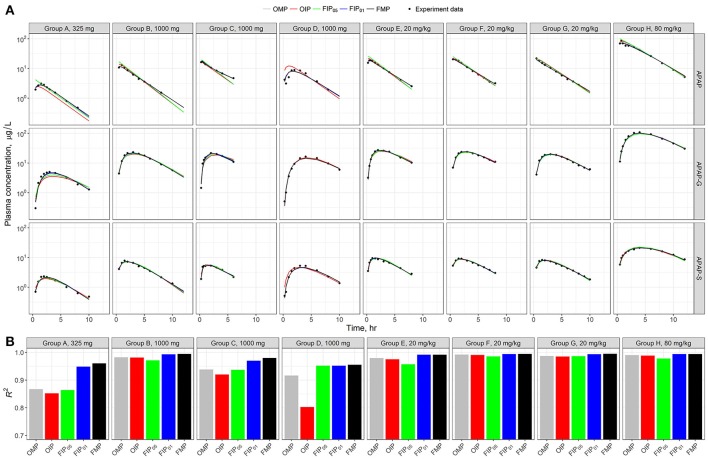
**(A)** Model evaluation results for the eight experimental human studies with different APAP dosages (Top) across the five parameter sets: original model parameters, OMP (gray); full set of model parameters, FMP (black); 11 original influential parameters, using 0.05 as a cut-off point, OIP (red); full set of 10 influential parameters, using 0.05 as a cut-off point, FIP_05_ (green); and full set of 20 influential parameters, using 0.01 as a cut-off point, FIP_01_ (blue). **(B)** The *R*^2^ was used to assess the model performance for each experimental group.

Figure 5A shows the model time-course predictions across the different analyses for each human subject study group separately. Visual inspection of the data points relative to the scatter of the predictions suggests that each parameter set shows a consistent or similar predicted curve in the high-dosage (20 and 80 mg/kg) groups (E–H). The low-dose groups (325 and 1,000 mg) (A–D) showed slightly different calibration results in the predicted curves from the given parameter set. To better quantify and characterize the model performance and predicated differences, we used the coefficient of determination (*R*^2^) as a metric of precision (Figure 4B). Results show that the estimated R^2^ were relatively high in all simulation sets (*R*^2^ > 0.7). Across all the different analyses, the best performance was from the FMP and the “influential” parameters FIP_01_ (all estimated *R*^2^ > 0.9)—higher than the results from the OMP, OIP, or FIP_05_.

### Does fixing parameters using “expert judgment” lead to unintentional imprecision or bias?

**Figure 6:**
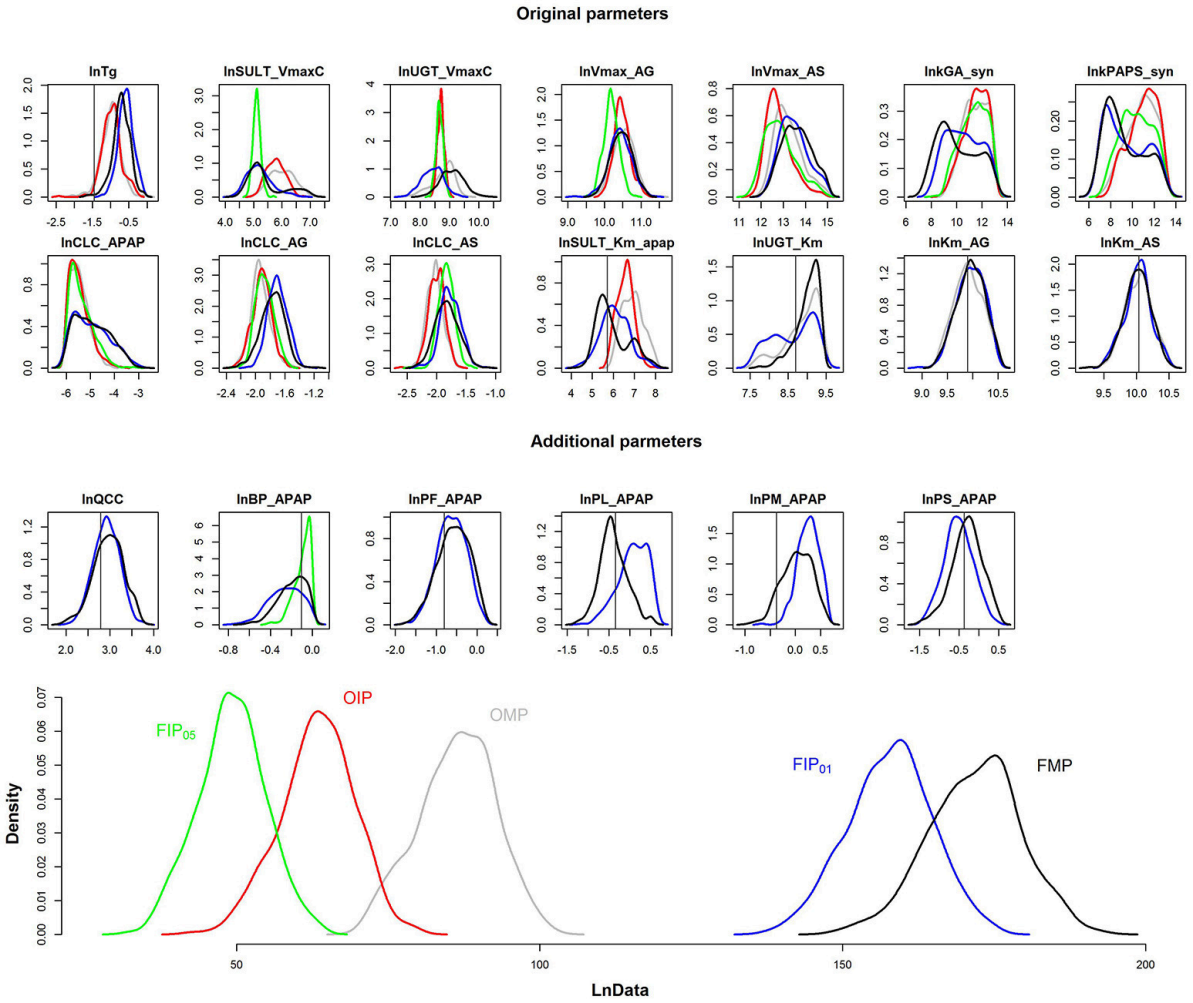
Comparison of the marginal posterior distributions of influential parameters and log-likelihood (LnData) for OMP (gray), OIP (red), FMP (black), FIP_05_ (green), FIP_01_ (blue). The vertical line represents the prior mean and nominal value of original and additional parameters, respectively.

The results depicted in Figures 4, 5 illustrate that GSA could identify “influential” subsets of parameters that lead to pharmacokinetic predictions that are of similar or greater precision and accuracy than predictions using subsets of parameters identified by “expert judgment.” Figure 6 shows the comparison of the marginal posterior distributions for 20 influential parameters in FIP_01_. Some parameters showed similar distributions among different analyses, such as the hepatic transporter constants (Km_AG and Km_AS), the cardiac output (QCC), and the partition coefficient of fat (PF_APAP). Moreover, the fixed values of QCC and PF_APAP in the OMP were similar to the central estimates of the posterior distributions in the analyses based on the FMP. However, for some parameters, such as the partition coefficient of muscle (PM_APAP), the fixed nominal value was closer to the tail of the posterior distribution when these parameters were estimated. *Thus, fixing parameters using “expert judgment” can lead to bias in some of the parameter estimates*.

Figure 6 is the posterior distribution of the LnData across the different analyses. For OMP and OIP, the log-likelihood distributions overlapped, indicating similar model fit. The log-likelihood distribution for FIP_05_ was substantially below both the OMP and FMP. However, for FIP_01_, using the cut-off of 0.01, not only did the log-likelihood distribution overlap with FMP, based on all the parameters, but it was also substantially greater than the log-likelihood using the OMP. *Thus, GSA was more effective than “expert judgment” at identifying parameters that are influential, and led to a better fit between predictions and data even though almost the same number of parameters were used (20 vs. 21)*.

## Discussion

### Efficiency of GSA algorithms

Similar to results from previous studies, we found that the Morris method showed the least computational burden compared to several other approaches used in GSA. Among the variance-based methods, the three methods we tested gave highly correlated results, but with different computational burdens and rates of convergence. For the same number of samples, the Owen method had the highest computational burden, with eFAST and Jansen being similar. However, eFAST required the least number of samples to reach convergence, with the Jansen and Owen methods sometimes not reaching convergence even after 10 h of wall-clock time. This is likely because Owen and Jansen make use of multiple independent Monte Carlo samples that increase the number of model evaluations and computational demands. Consistent with previous analyses (Sarrazin et al., 2016), we found no clear predictive relationship between the number of parameters and the sample number needed to reach reasonable convergence, other than that more computational time is needed to complete the analysis for a larger number of model parameters.

Unlike the previous analyses by McNally et al. (2011) and Loizou et al. (2015), we found that the Morris method was not always effective at screening or ranking parameters, even with proper normalization. While ranking of parameters was fairly consistent between Morris and Sobol indices, for screening, the Morris index was able to eliminate few if any parameters as non-influential. Specifically, considering both main and interaction effects, the Morris method identified all 21 OMP as influential and all but three of the 58 FMP as influential. Only a few other studies have compared and discussed the relationship between these two indices (Campolongo et al., 2007; Confalonieri et al., 2010; Herman et al., 2013; Vanrolleghem et al., 2015). Campolongo et al. (2007) and Herman et al. (2013) found that the results of the Morris method compared favorably with the results of variance-based sensitivity methods. However, Confalonieri et al. (2010) indicated that Morris and variance-based method might give different results in parameter ranking when the nonlinearity exists in the model. More concerning is that Vanrolleghem et al. (2015) found that Morris method could overestimate the number of non-influential model factors compared to the eFAST method, potentially leading to improper “fixing” of influential parameters. Our results are intermediate between these, in that there some “false positives” with respect to influential parameters, but not any “false negatives,” which would be of greater concern.

Our results are similar to Herman et al. (2013), which indicated that the relationships between Morris μ^*^ and Sobol indices are approximately linear for the low-sensitivity parameters, but nonlinear for the high-sensitivity parameters. Also, Herman et al. (2013) found that the rank correlation (Spearman) shows more consistency between the Morris and Sobol indices than the product moment correlation (Pearson). Given the computational efficiency of the eFAST algorithm (requiring less than 10 min when using the R “sensitivity” package and GNU MCSim), the use of the Morris method as an initial screen could be limited to cases where model evaluations are substantially more computationally burdensome.

### Distinguishing influential and non-influential parameters

Several previous studies applied GSA in the calibration of the complex models, such as environmental and biological models. However, ranking of parameter sensitivities is the usual method in the GSA approach (Boas et al., 2015), and most of the past studies did not provide specific criteria that could be used to distinguish influential and non-influential parameters. Additionally, some studies only focused on the variation in the steady state or integrated measures (e.g., area under the curve) rather than full time-course behaviors (Safta et al., 2015; Zhang et al., 2015). Consistent with the work of McNally et al. (2011), our study found that parameter sensitivity can change with time and different output variables. Using a global approach based on a heatmap visualization combined with an index “cut-off,” we could systematically distinguish between “influential” and “non-influential” parameters. In our case, a cut-off of 0.01 for the FMP and a cut-off of 0.05 for the OMP worked as a compromise between efficiency, accuracy, and precision.

### Increasing efficiency of bayesian PBPK model calibration without compromising accuracy

Bayesian analysis is a statistically-rigorous approach to address the challenges of calibrating PBPK models. By incorporating prior information, Bayesian posterior inference is valid even with non-identifiable parameters (Tsamandouras et al., 2015). However, the computational cost of MCMC algorithms, even with modern fast computers, has limited the application of Bayesian approaches in drug development and evaluation (Langdon et al., 2007; Gibiansky et al., 2012). Therefore, except for simple models, the general practice is to fix a certain subset of parameters based on “expert judgment” to enable convergence in a reasonable timeframe. However, it is not clear whether “expert judgment” leads to compromises in terms of accuracy or precision. Our hypothesis was that using GSA to decide between parameters that needed to be estimated and those that could be fixed would lead to increased efficiency without leading to bias. Indeed, we found that using only “influential” parameters in model calibration had little discernable effect on prediction accuracy and precision, while substantially reducing the computational burden. Additionally, by conducting GSA on the FMP, including those that were previously fixed, we identified a subset of parameters that, when calibrated, lead to improved model fit compared to the OMP. Moreover, some previously fixed parameters had posterior distributions that were shifted away from their nominal values. In sum, our results indicate that GSA can improve the efficiency of Bayesian PBPK model calibration relative to traditional techniques for parameter selection based on expert judgment without compromising accuracy.

### Limitations and future research

In our sensitivity analysis, all parameters' mean values and ranges were based on published references when available. We defined the ranges of the parameter distributions to be two to three times the standard deviation given in these references. During our testing, we found that this range had an influence on the sensitivity measures of each parameter and is, therefore, an important future area of exploration for GSA studies. A notable limitation of the present analysis is that the approach was demonstrated using a single PBPK model, and therefore the degree to which the results can be generalized was not established. However, we do not expect that results for different PBPK models and data will differ with respect to different GSA algorithms and their computational efficiency; though, the appropriate “cut-off” points for distinguishing between “influential” and “non-influential” parameters may differ for different models and datasets. Nonetheless, in the future, additional case studies should be conducted to verify that specific values proposed in this study can be used in general. Moreover, we made the assumption that the input parameters in the PBPK model are independent in the current study. Based on recent work by Ring et al. (2017) that compared PBPK modeling results with and without correlations, the impact of this assumption is likely to be small. However, further investigation of the impacts of correlations could be performed using approaches such as that by Li et al. (2010).

## Conclusion: an updated GSA-based workflow for PBPK modeling

In this study, we developed an approach to apply GSA to reduce the computational burden in the Bayesian, MCMC-based calibration process of a PBPK model. In comparing the EE-based Morris method and three variance-based sensitivity methods (eFAST, Jansen, and Owen), we found that the eFAST method had the best balance of efficiency and accuracy. We found that applying the eFAST approach for a complex multi-compartment, multi-dataset, multi-metabolite PBPK model required less than 10 min of computational time on current hardware. We also developed a “cut-off”-based approach to consistently distinguish between “influential” and “non-influential” parameters. Finally, we demonstrated that performing Bayesian model calibration using only “influential” parameters, and fixing “non-influential” parameters, can lead to greater efficiency without compromising accuracy. The GSA-based approach was shown to be more reliable than an “expert judgment”-based approach to fixing parameters.

Our results suggest the following efficient workflow for applying GSA to Bayesian PBPK model calibration:

1. Establish prior distributions for all parameters, and ensure that the prior predictions cover the range of data being used for model calibration.
2. Only if model evaluation is computationally burdensome, use the Morris method as an initial screen to remove clearly “non-influential” parameters, making sure to normalize separately for each output as well as to check convergence.
3. Use the eFAST method for parameter sensitivity, making sure to check convergence using the method of Sarrazin et al. (2016).
4. Visualize parameter sensitivity using a “heatmap” approach, distinguishing “influential” and “non-influential” parameters with a cut-off such as 0.01 or 0.05 (for Sobol indices) or 0.1 (for normalized Morris indices), so that any parameter with a sensitivity index for at least one output greater than the cut-off would be identified as “influential.” The cut-off approach to identify and classify parameters could also be implemented in software once reasonable threshold values are established.
5. Conduct model calibration using MCMC simulation for only the “influential” parameters, fixing “non-influential” parameters at nominal values.

We expect that routinely implementing such a workflow will enable broader application and use of Bayesian PBPK model calibration by substantially reducing its associated computational burden and allowing for greater ranges of studies within a given amount of resources. Overall, we have focused on one particular use of GSA—dimensionality reduction and computational efficiency. However, in combination with expert judgment, GSA is also a very useful approach for identifying coding errors in the model.

## Author contributions

N-HH, BR, FB, and WC are listed as authors. WC conceived the overall research concept and design. N-HH contributed to the research design, had overall responsibility for its implementation, and performed all analyses, simulations, and their interpretation. N-HH and WC prepared the initial draft of the manuscript. BR provided the original PBPK model and data. FB contributed to the implementation of MCMC simulations. All authors reviewed the manuscript and revised it critically.

### Conflict of interest statement

The authors declare that the research was conducted in the absence of any commercial or financial relationships that could be construed as a potential conflict of interest.
