# Supplementary material for: Applying a Global Sensitivity Analysis Workflow to Improve the Computational Efficiencies in Physiologically-Based Pharmacokinetic Modeling
Source: Front Pharmacol. 2018 Jun 8;9:588. doi: 10.3389/fphar.2018.00588 (PMC6002508; doi:10.3389/fphar.2018.00588)
Supplement: Supplementary file 1 [file Table_1.PDF]

Supplementary Material

Applying A Global Sensitivity Analysis Workflow to Improve the Computational Efficiencies in Physiologically-Based Pharmacokinetic Modeling

Nan-Hung Hsieh [1], Brad Reisfeld [2], Frederic Y. Bois [3], Weihsueh A. Chiu [1]*

*** Correspondence:** Weihsueh A Chiu: wchiu@cvm.tamu.edu

1 Supplementary Figures and Tables

Supplementary Table 1. Summary of the human experiments used in this study

Group	Dose	No. of subjects	Reference
1	325 mg	8	(Volak et al., 2013)
2	1000 mg	6	(Jensen et al., 2004)
3	1000 mg	5	(Shinoda et al., 2007)
4	1000 mg	12	(Kim et al., 2011)
5	20 mg/kg	8	(Prescott, 1980)
6	20 mg/kg	6	(Chan et al., 1997)
7	20 mg/kg	11	(Critchley et al., 1986)
8	20 mg/kg	9	(Chiew et al., 2010)

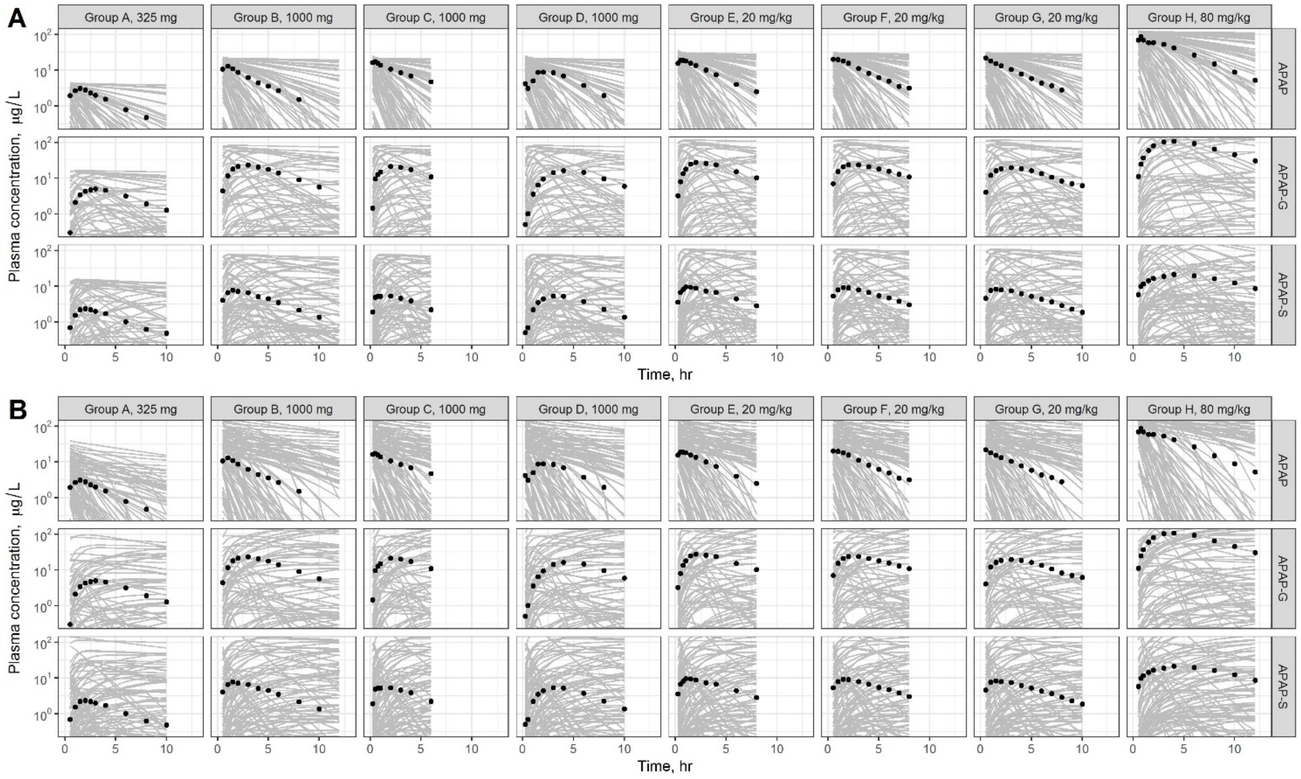

Supplementary Figure 1. Coverage checks of prior PBPK model predictions with calibration data for (A) original model parameters (OMP) and (B) full set of model parameters (FMP).

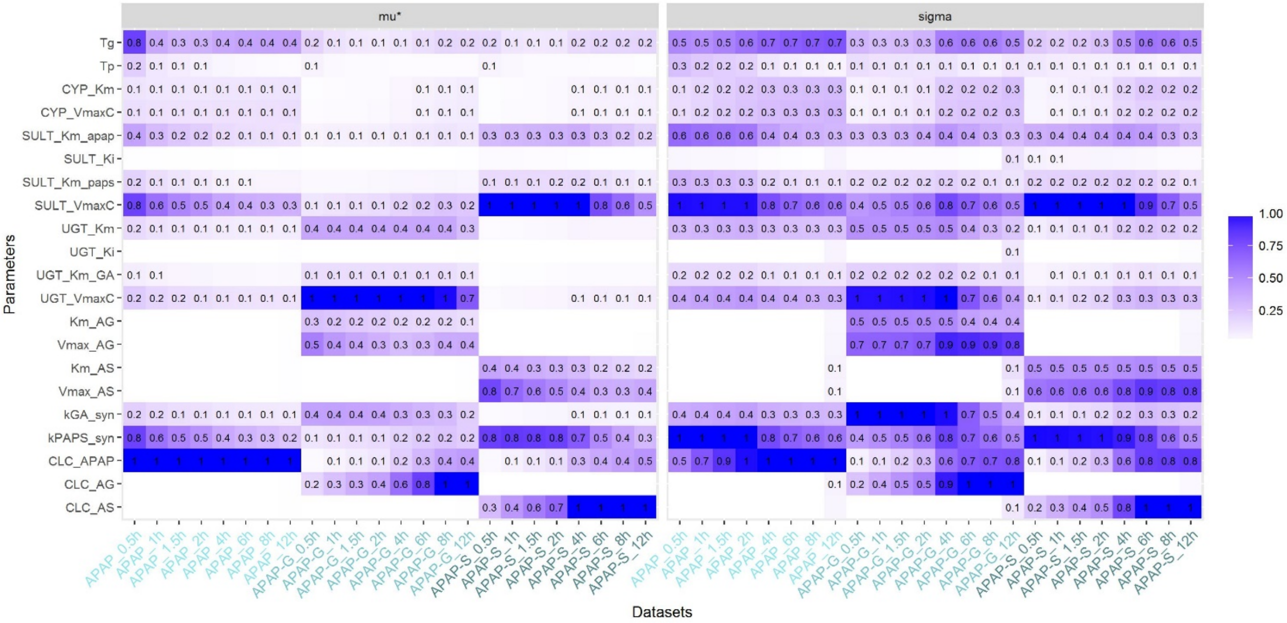

Supplementary Figure 2. Heat map representation of time-dependent normalized sensitivity coefficients computed using the Morris method (Morris, 1991), for the original model parameters (OMP). The displayed indices are normalized relative to the maximum estimated value, and not displayed for values <0.1.

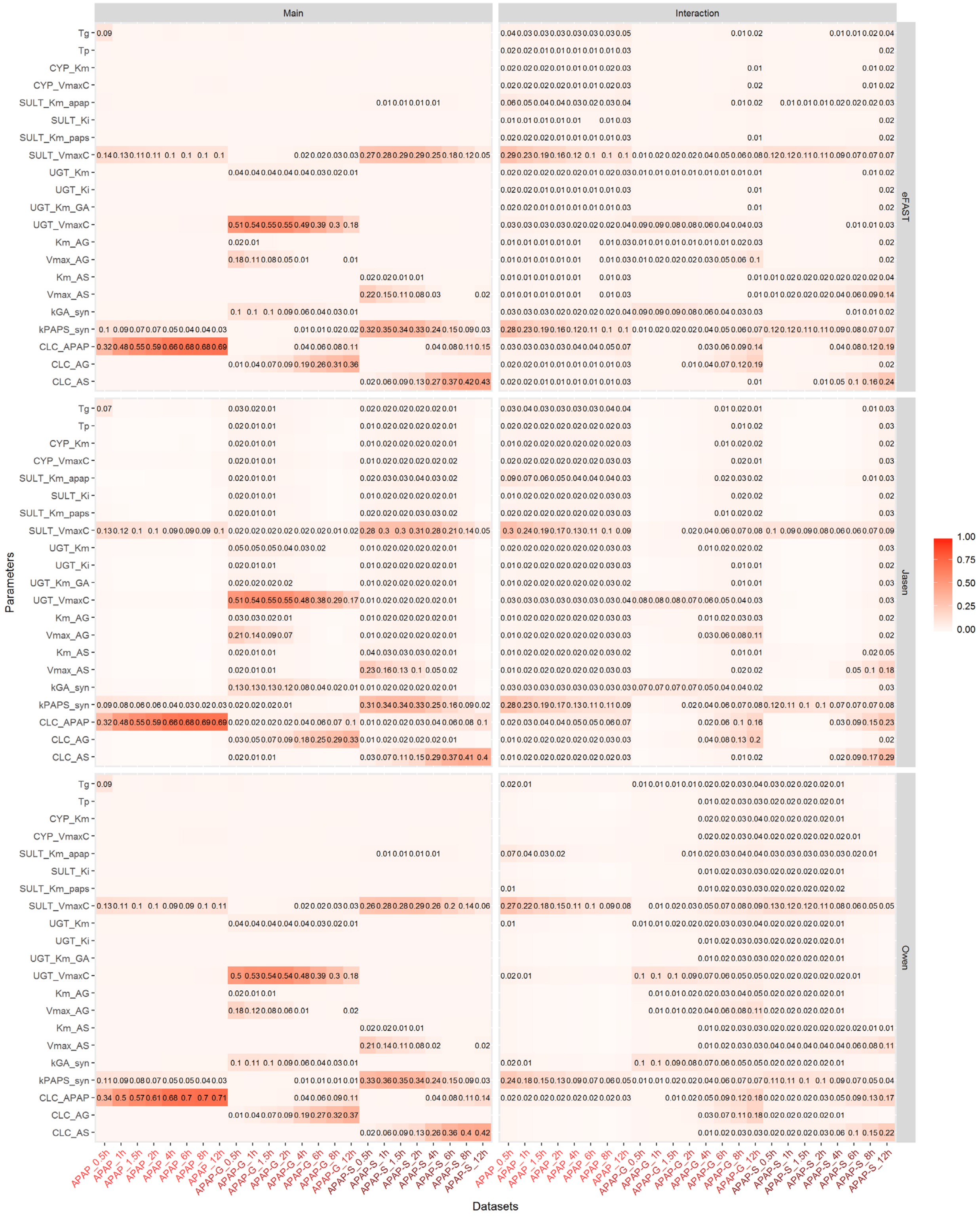

Supplementary Figure 3. Heat map representation of time-dependent sensitivity coefficients computed for GSA method [eFAST (Saltelli et al., 1999), Jansen (Jansen, 1999), and Owen (Owen, 2013)] with original model parameters (OMP). The indices are not displayed for values <0.01.

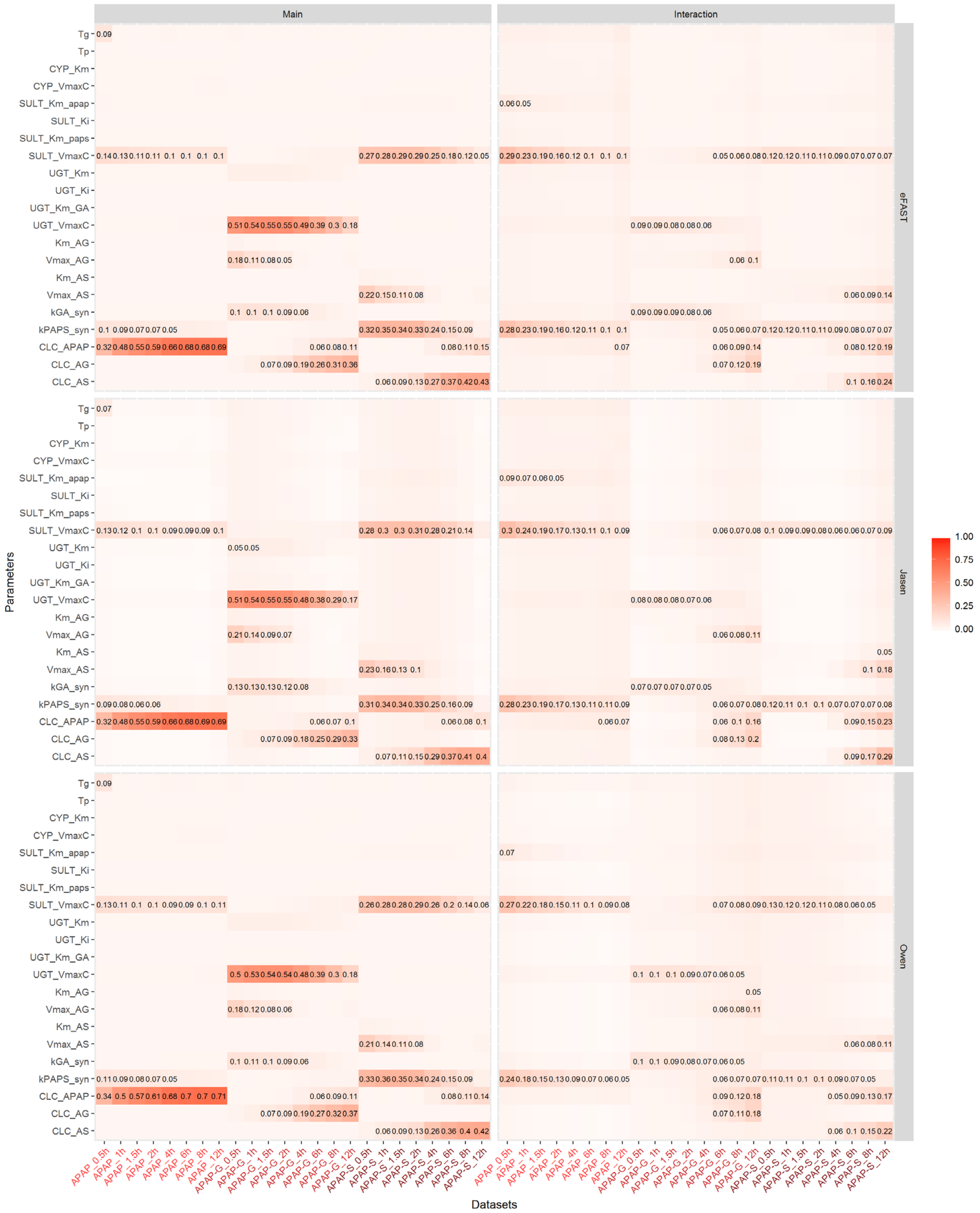

Supplementary Figure 4. Heat map representation of time-dependent sensitivity coefficients computed for GSA method [eFAST (Saltelli et al., 1999), Jansen (Jansen, 1999), and Owen (Owen, 2013)] with original model parameters (OMP). The indices are not displayed for values <0.05.

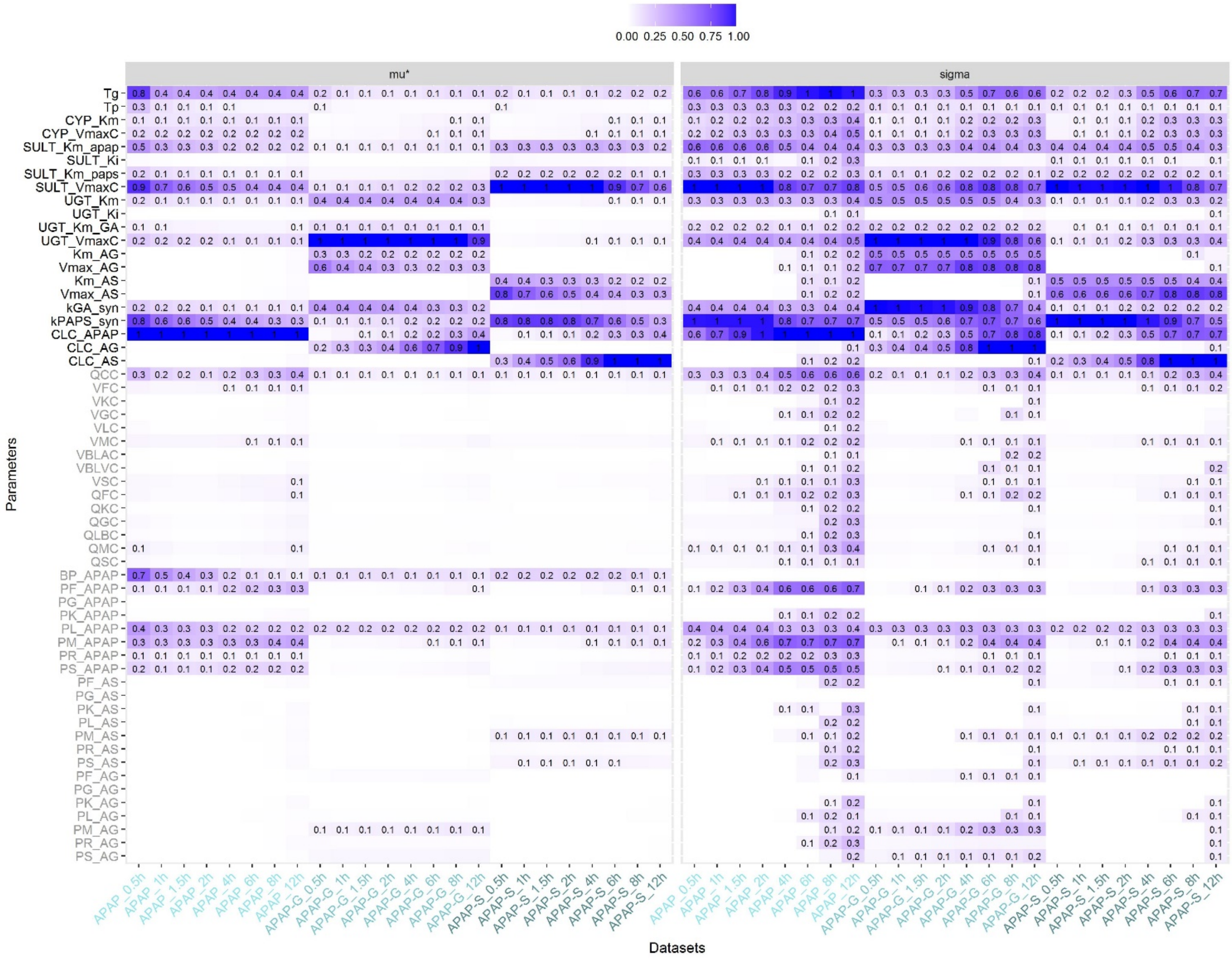

Supplementary Figure 5. Heat map representation of time-dependent normalized sensitivity coefficients computed using the Morris method (Morris, 1991), with the full set of model parameters (FMP). The displayed indices are normalized relative to the maximum estimated value, and not displayed for values <0.1. Original and new parameters were shown in black and grey text, respectively.

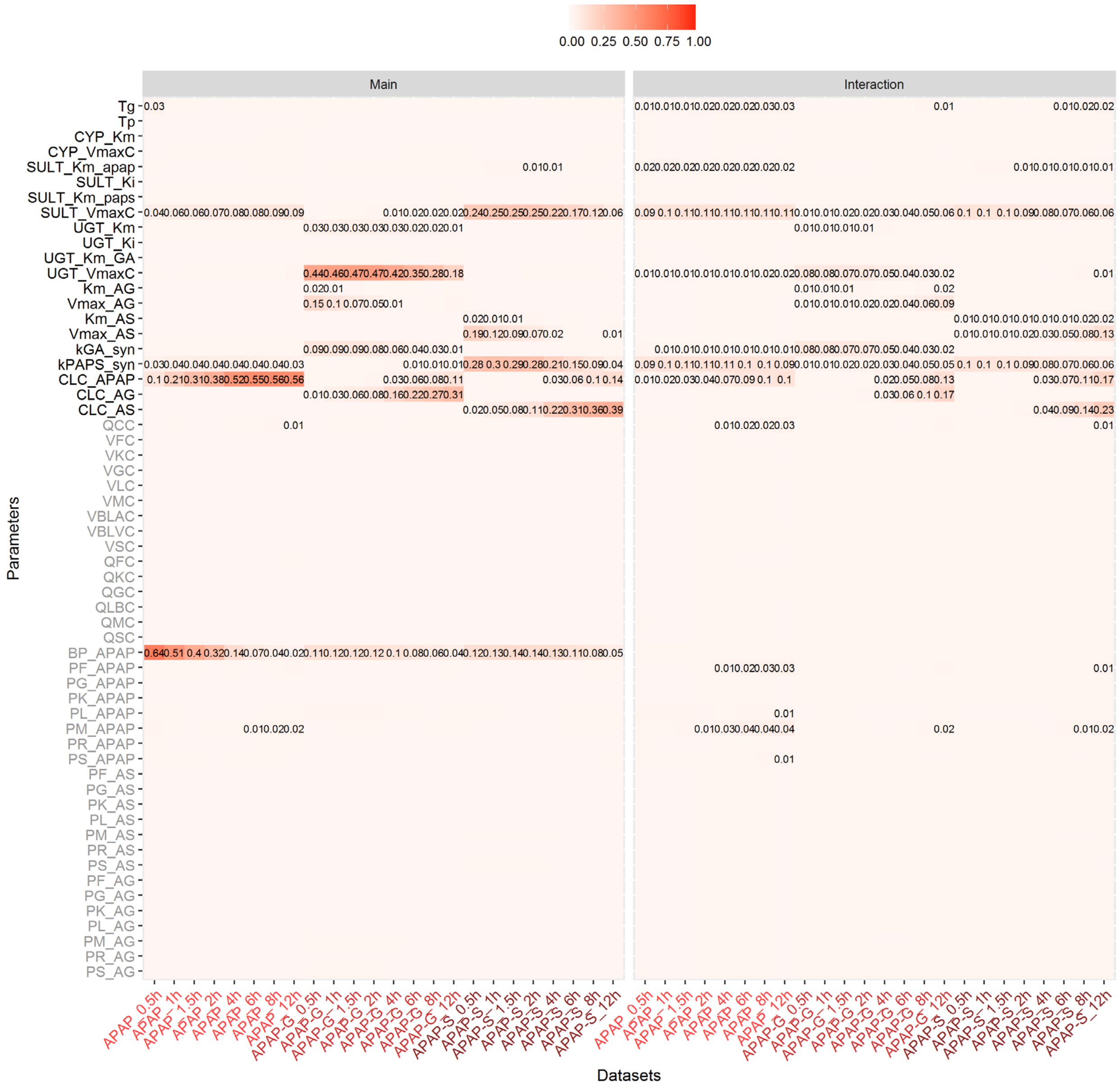

Supplementary Figure 6. Heat map representation of time-dependent sensitivity coefficients computed for eFAST (Saltelli et al., 1999) with the full set of parameters (FMP). The indices are not displayed for values <0.01. Original and new parameters were shown in black and grey text, respectively.

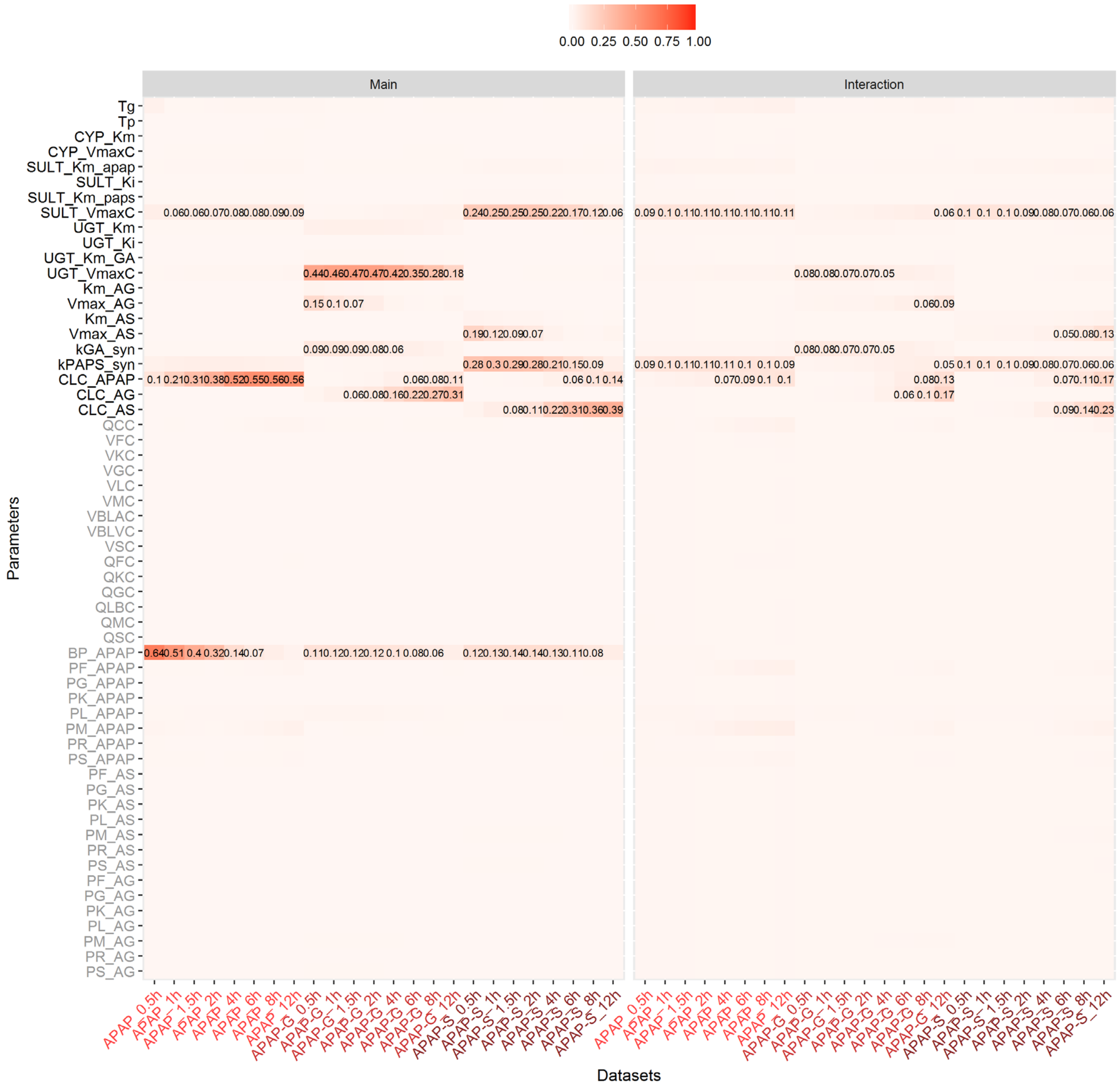

Supplementary Figure 7. Heat map representation of time-dependent sensitivity coefficients computed for eFAST (Saltelli et al., 1999) with the full set of parameters (FMP). The indices are not displayed for values <0.05. Original and new parameters were shown in black and grey text, respectively.

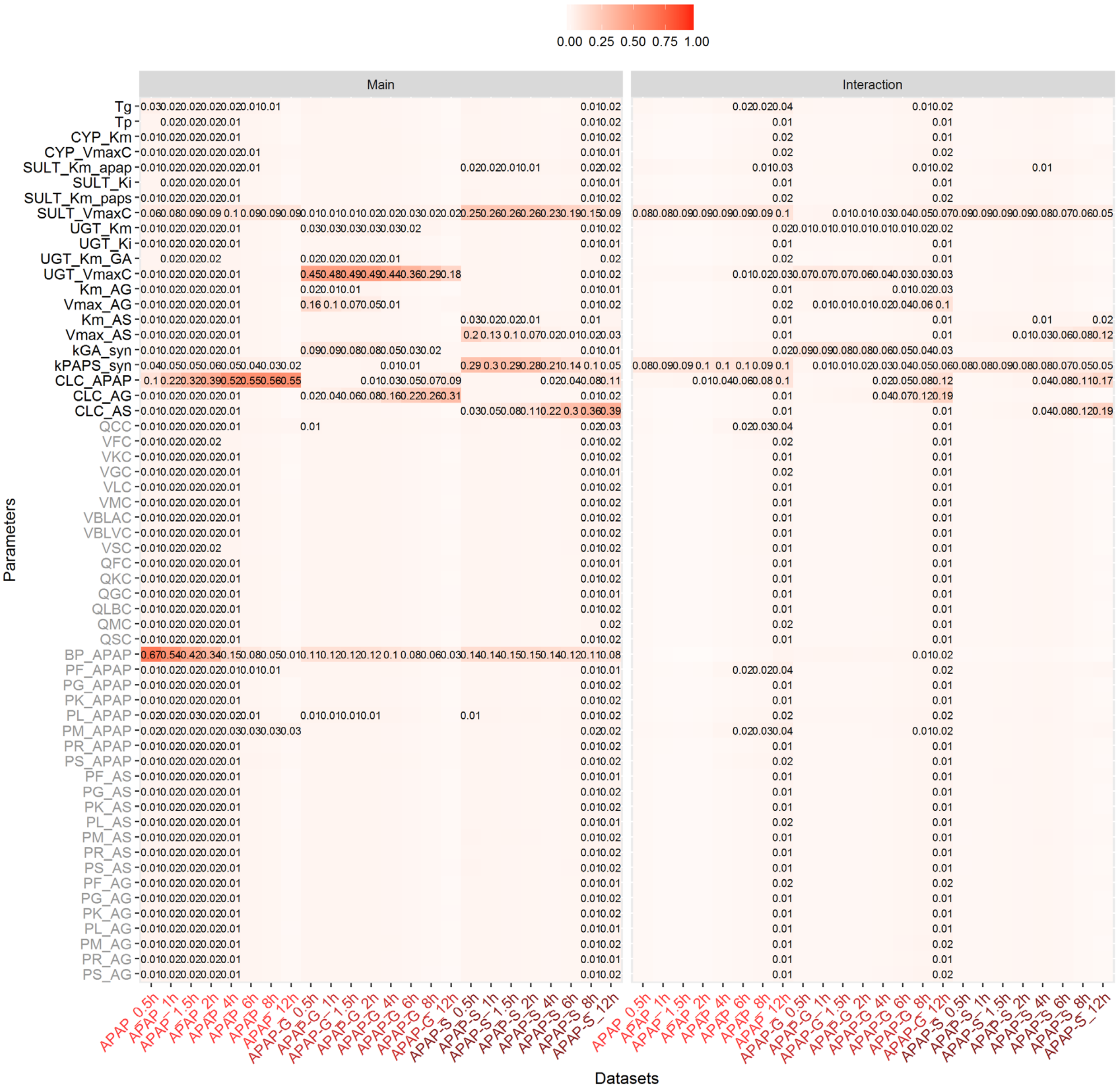

Supplementary Figure 8. Heat map representation of time-dependent sensitivity coefficients computed for Jansen (Jansen, 1999) with the full set of parameters (FMP). The indices are not displayed for values <0.01. Original and new parameters were shown in black and grey text, respectively.

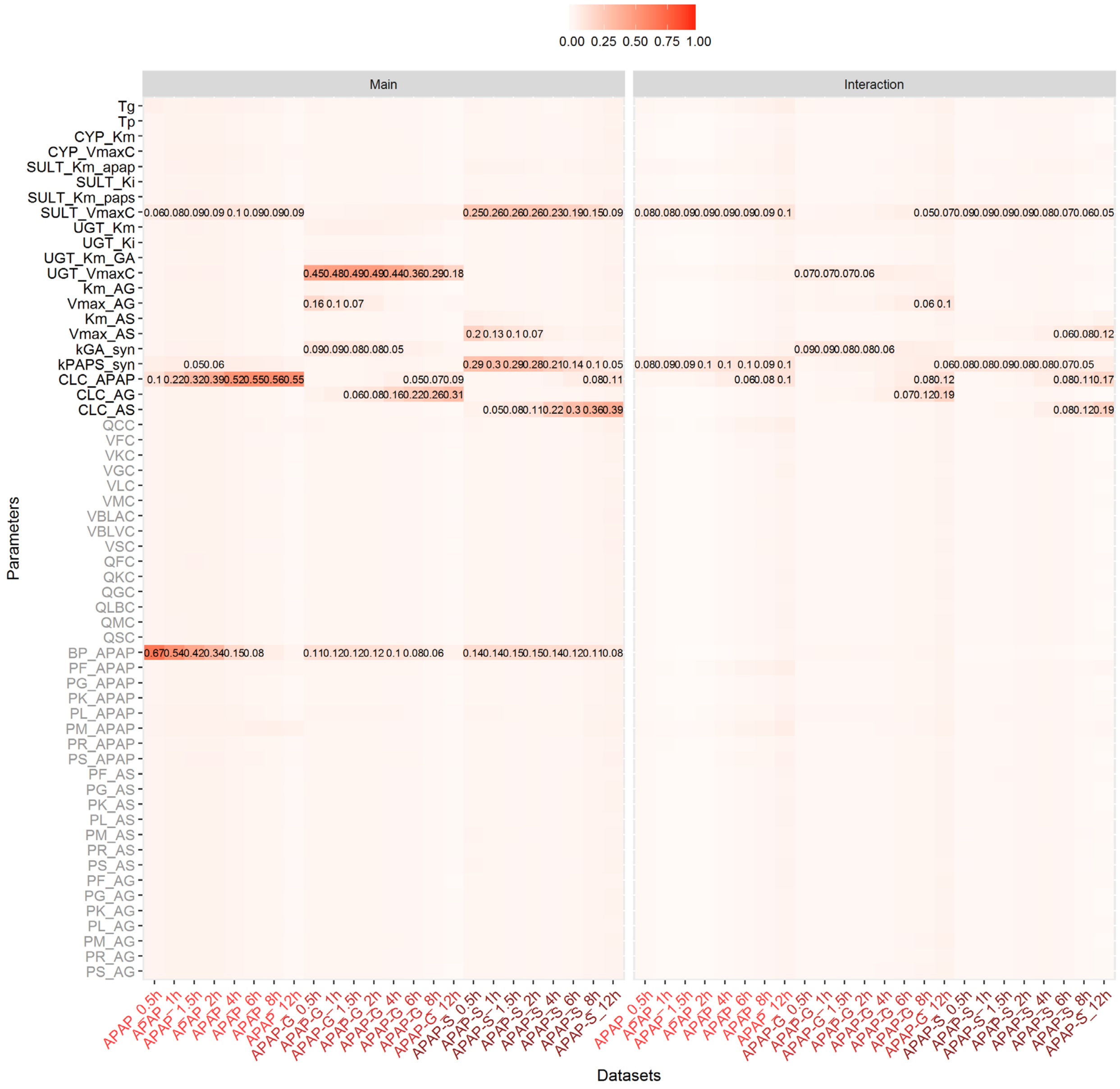

Supplementary Figure 9. Heat map representation of time-dependent sensitivity coefficients computed for Jansen (Jansen, 1999) with the full set of parameters (FMP). The indices are not displayed for values <0.05. Original and new parameters were shown in black and grey text, respectively.

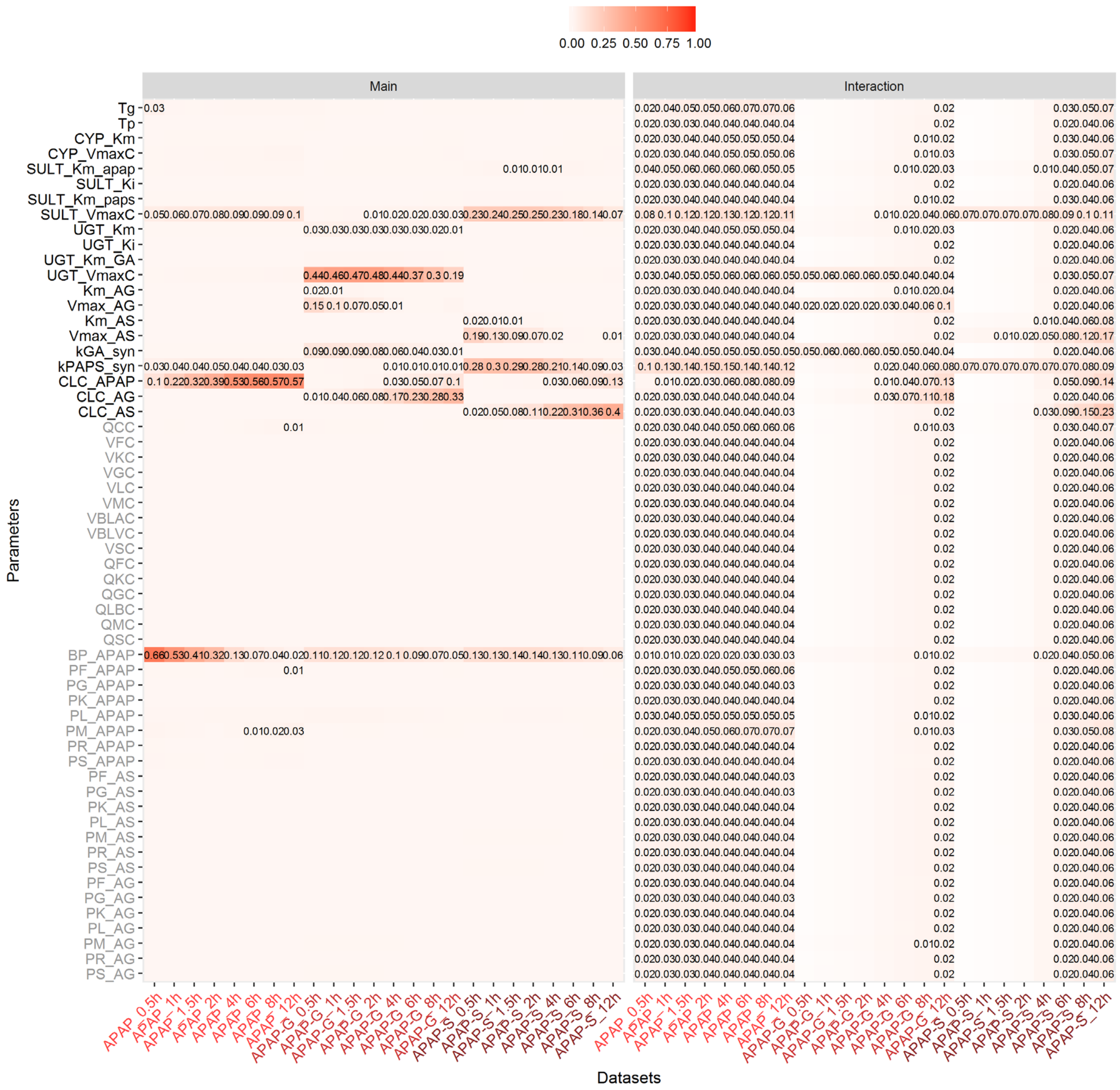

Supplementary Figure 10. Heat map representation of time-dependent sensitivity coefficients computed for Owen (Owen, 2013) with the full set of parameters (FMP). The indices are not displayed for values <0.01. Original and new parameters were shown in black and grey text, respectively.

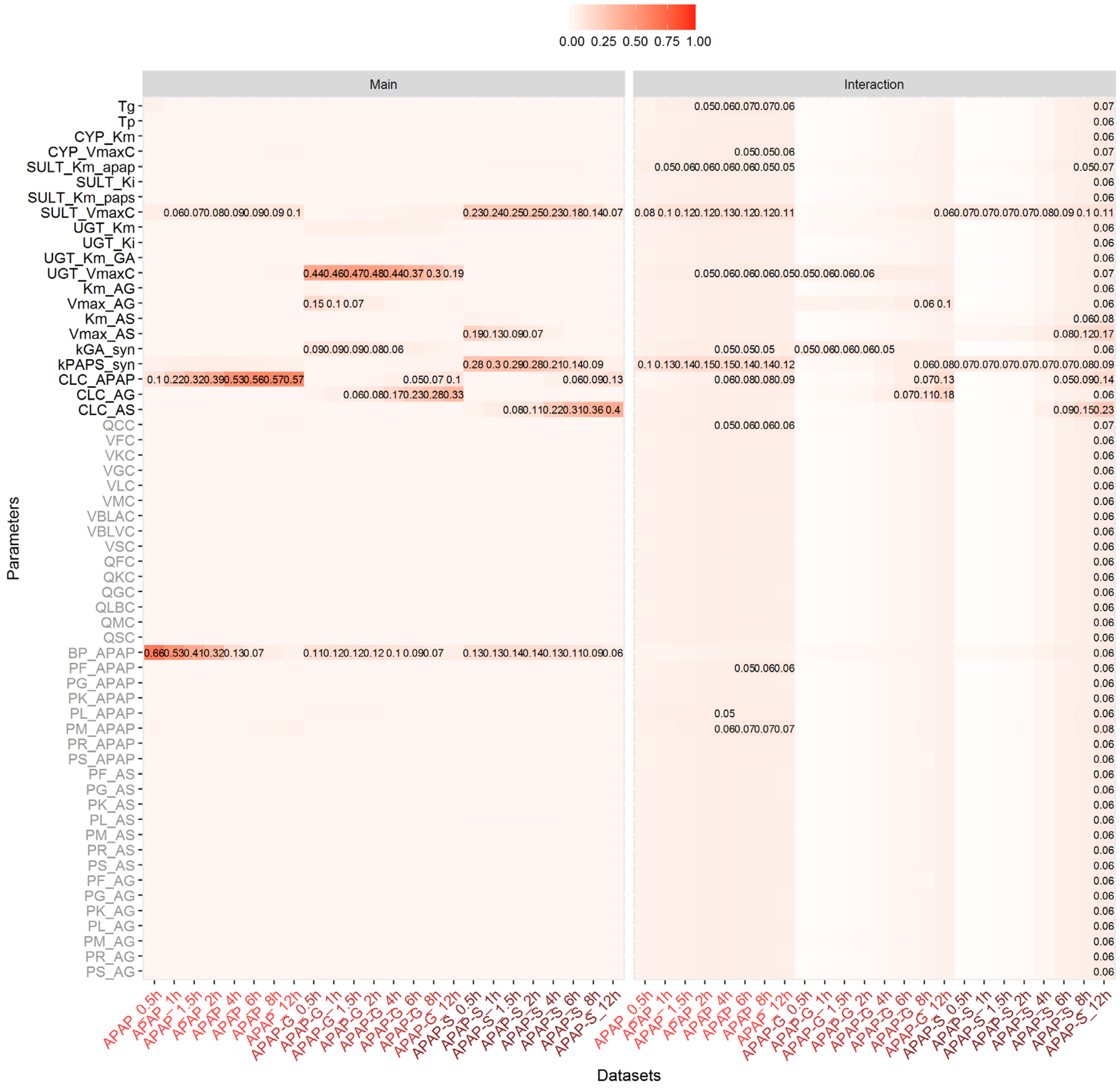

Supplementary Figure 11. Heat map representation of time-dependent sensitivity coefficients computed for Owen (Owen, 2013) with the full set of parameters (FMP). The indices are not displayed for values <0.01. Original and new parameters were shown in black and grey text, respectively.

References

Chan, M. T., Anderson, P. J., Chan, J. C., Lau, G. S., and Critchley, J. A. (1997). Single-dose pharmacokinetics of paracetamol and its conjugates in Chinese non-insulin-dependent diabetic patients with renal impairment. *Eur. J. Clin. Pharmacol.* 52, 285–288.

Chiew, A., Day, P., Salonikas, C., Naidoo, D., Graudins, A., and Thomas, R. (2010). The comparative pharmacokinetics of modified-release and immediate-release paracetamol in a simulated overdose model. *Emerg. Med. Australas.* 22, 548–555. doi:10.1111/j.1742-6723.2010.01354.x.

Critchley, J. A., Nimmo, G. R., Gregson, C. A., Woolhouse, N. M., and Prescott, L. F. (1986). Inter-subject and ethnic differences in paracetamol metabolism. *Br. J. Clin. Pharmacol.* 22, 649–657.

Jansen, M. J. W. (1999). Analysis of variance designs for model output. *Comput. Phys. Commun.* 117, 35–43. doi:10.1016/S0010-4655(98)00154-4.

Jensen, L. S., Valentine, J., Milne, R. W., and Evans, A. M. (2004). The quantification of paracetamol, paracetamol glucuronide and paracetamol sulphate in plasma and urine using a single high-performance liquid chromatography assay. *J. Pharm. Biomed. Anal.* 34, 585–593.

Kim, D.-W., Tan, E. Y., Jin, Y., Park, S., Hayes, M., Demirhan, E., et al. (2011). Effects of imatinib mesylate on the pharmacokinetics of paracetamol (acetaminophen) in Korean patients with chronic myelogenous leukaemia. *Br. J. Clin. Pharmacol.* 71, 199–206. doi:10.1111/j.1365-2125.2010.03810.x.

Morris, M. D. (1991). Factorial sampling plans for preliminary computational experiments. *Technometrics* 33, 161–174. doi:10.2307/1269043.

Owen, A. B. (2013). Better estimation of small Sobol' sensitivity indices. *ACM Trans Model Comput Simul* 23, 11:1–11:17. doi:10.1145/2457459.2457460.

Prescott, L. F. (1980). Kinetics and metabolism of paracetamol and phenacetin. *Br. J. Clin. Pharmacol.* 10 Suppl 2, 291S-298S.

Saltelli, A., Tarantola, S., and Chan, K. P.-S. (1999). A quantitative model-independent method for global sensitivity analysis of model output. *Technometrics* 41, 39–56. doi:10.2307/1270993.

Shinoda, S., Aoyama, T., Aoyama, Y., Tomioka, S., Matsumoto, Y., and Ohe, Y. (2007). Pharmacokinetics/pharmacodynamics of acetaminophen analgesia in Japanese patients with chronic pain. *Biol. Pharm. Bull.* 30, 157–161.

Volak, L. P., Hanley, M. J., Masse, G., Hazarika, S., Harmatz, J. S., Badmaev, V., et al. (2013). Effect of a herbal extract containing curcumin and piperine on midazolam, flurbiprofen and paracetamol (acetaminophen) pharmacokinetics in healthy volunteers. *Br. J. Clin. Pharmacol.* 75, 450–462. doi:10.1111/j.1365-2125.2012.04364.x.
